# Effects of a Multidisciplinary Intervention on Fatigue in Lymphoma Survivors With Chronic Fatigue: Protocol for a Randomized Controlled Trial (REFUEL)

**DOI:** 10.2196/69336

**Published:** 2025-08-29

**Authors:** Synne-Kristin Hoffart Bøhn, Kristin V Reinertsen, Cecilie E Kiserud, Jon Håvard Loge, Alexander Fosså, Tone Skaali, Rune Blomhoff, Line Merethe Oldervoll, Kerry S Courneya, Truls Raastad, Tormod S Nilsen, Torbjørn Wisløff, Hanne Cathrine Lie, Torkil Berge, Elisabeth Edvardsen, Unn-Merete Fagerli, Elin Fjerstad, Gunhild Maria Gjerset, Ingvild Haavik, Hege Berg Henriksen, Arkady Rutkovskiy, Gro Sandberg, Mette Seland, Malene Slott, Kristin Holm Tjessem, Linn Viktil, Lene Thorsen

**Affiliations:** 1 Department of Oncology Division of Cancer Medicine Oslo University Hospital Oslo Norway; 2 Department of Behavioural Medicine University of Oslo Oslo Norway; 3 Department of Clinical Service Division of Cancer Medicine Oslo University Hospital Oslo Norway; 4 Department of Nutrition University of Oslo Oslo Norway; 5 Centre for Crisis Psychology Faculty of Psychology University of Bergen Bergen Norway; 6 Department of Mental Health Norwegian University of Science and Technology Trondheim Norway; 7 Faculty of Kinesiology, Sport, and Recreation University of Alberta Edmonton, AB Canada; 8 Institute of Physical Performance Norwegian School of Sport Sciences Oslo Norway; 9 Health Services Research Unit Akershus University Hospital Lillestrøm Norway; 10 Department of Psychiatry Diakonhjemmet Hospital Oslo Norway; 11 Department of Pulmonary Medicine Oslo University Hospital Oslo Norway; 12 Department of Oncology St. Olavs Hospital, Trondheim University Hospital Trondheim Norway; 13 Department of Clinical and Molecular Medicine Norwegian University of Science and Technology Trondheim Norway; 14 Department of Cardiology Oslo University Hospital Oslo Norway

**Keywords:** cancer, lymphoma, late effects, chronic fatigue, patient education, physical exercise, cognitive behavioral therapy, nutritional counseling

## Abstract

**Background:**

Chronic fatigue (CF) is a highly disabling late effect after cancer, affecting 25% to 40% of lymphoma survivors years after cancer treatment. There is a lack of randomized controlled trials testing interventions to reduce fatigue levels among survivors with CF.

**Objective:**

The primary aim of the Randomized Controlled Trial in Chronically Fatigued Lymphoma Survivors (REFUEL) is to examine the effects of a multidisciplinary intervention on the level of fatigue among lymphoma survivors with CF before and immediately after the intervention (3 months after randomization). Secondary aims are to (1) investigate the effects of the intervention on the level of fatigue 6 and 9 months after randomization and the effects on health-related quality of life (HRQoL); other patient-reported outcome measures; and physical fitness 3, 6, and 9 months after randomization; (2) evaluate the cost-utility of the intervention; (3) examine the effect of the intervention on HRQoL among the survivors’ partners; and (4) investigate the long-term perceived benefits and daily use of acquired self-management strategies, as well as measure changes in fatigue, daily functioning, HRQoL, mental health, and work-life balance management within each group at 1- and 2-year follow-ups.

**Methods:**

The REFUEL trial is a 2-armed randomized controlled trial. Lymphoma survivors (2-12 years after diagnosis) with CF are randomly allocated to a 12-week multidisciplinary intervention including patient education, physical exercise, a cognitive behavioral therapy–based group program, and individual nutrition counseling or to usual care. Fatigue is measured by the Chalder Fatigue Questionnaire. Other patient-reported outcome measures are measured by validated questionnaires (eg, the European Organization for Research and Treatment of Cancer Core Quality of Life Questionnaire, the Patient Health Questionnaire-9, and the Generalized Anxiety Disorder-7). Cardiorespiratory fitness is measured as peak oxygen consumption during a cardiopulmonary exercise test or indirectly using a modified Balke treadmill protocol. Muscle strength is assessed by push-ups and leg press. The primary analyses will be performed using a generalized linear mixed model for repeated measures, with an intention-to-treat approach.

**Results:**

A total of 150 survivors were included from December 2021 to March 2023. Three-month postrandomization assessments were completed in June 2023 and 2-year follow-up assessments were completed in June 2025.

**Conclusions:**

The REFUEL trial will provide new and highly needed scientific evidence about the effects of a multidisciplinary intervention on the level of fatigue and secondary outcomes regarding HRQoL aspects among lymphoma survivors with CF.

**Trial Registration:**

ClinicalTrials.gov NCT05130099; https://www.clinicaltrials.gov/study/NCT05130099

**International Registered Report Identifier (IRRID):**

DERR1-10.2196/69336

## Introduction

### Background

Cancer-related fatigue, defined as “a subjective sense of physical, emotional, and/or cognitive tiredness or exhaustion related to cancer or cancer treatment that is not proportional to recent activity and interferes with usual functioning,” is one of the most common and disabling symptoms occurring during and after cancer treatment [1,2]. If the symptom persists for 6 months or more, it is defined as chronic fatigue (CF) [3].

As lymphoma is often diagnosed at a young age and has a high cure rate, survivors have long life expectancies after extensive treatments [4]. Thus, they are at risk for a range of late effects, including CF [4-10], which affects 25% to 42% of lymphoma survivors several years after treatment [9-12]. CF causes distress and limitations in health-related quality of life (HRQoL) domains, including social participation and family and work life [13,14]. It may also place a considerable economic burden on the society due to reduction or loss of employment and health care costs [15,16]. Furthermore, CF often reduces the ability to perform daily responsibilities and life roles at home [13]. Therefore, partners and family caregivers may provide considerable amounts of practical and emotional support in daily life, which may influence their own HRQoL [17,18].

The etiology of CF remains complex and unclear, and no effective pharmacological treatments are available [19,20]. However, CF is associated with several modifiable behavioral factors, including low cardiorespiratory fitness, psychological distress, and obesity [10,20-22]. No previous study has examined whether a multidisciplinary intervention targeting combinations of these factors is effective in reducing fatigue among survivors with CF, which seems logical due to the complexity of the symptom.

So far, most studies have tested the effects of physical exercise or psychological interventions on cancer-related fatigue during and shortly after cancer treatment [19,23-25]. A few intervention studies also indicate that diets rich in fruits, vegetables, whole grains, and anti-inflammatory fatty acids may improve fatigue [26]. This implies that future trials may add nutritional counseling to exercise and psychological interventions [26].

Meta-analyses, including those on exercise and psychological interventions, have shown limited effect sizes on fatigue [27,28]. This may in part be attributed to studies with nonfatigued participants or studies without fatigue as the primary outcome, and thus interventions not being specifically designed to reduce fatigue. Therefore, studies on long-term cancer survivors with CF are needed [1,19,25,29]. Moreover, studies evaluating effects of lifestyle interventions among cancer survivors on the HRQoL of the cancer survivors’ partners, and the cost-utility of such interventions, are lacking.

With this background, as well as our experiences from focus group interviews among cancer survivors with CF [30] and a feasibility study among lymphoma survivors [31], we planned a randomized controlled trial (RCT) in chronically fatigued lymphoma survivors (REFUEL). To the best of our knowledge, this is the first RCT to examine the effects of a multidisciplinary intervention, including patient education, physical exercise, a cognitive behavioral therapy (CBT)–based group program, and nutritional counseling on fatigue levels among lymphoma survivors experiencing fatigue years after treatment completion.

### Aims

The primary aim of the REFUEL trial is to examine the effect of a multidisciplinary intervention on the level of total fatigue before and immediately after the intervention (3 months after randomization) compared to usual care among lymphoma survivors with CF. Secondary aims are to examine the effects of the intervention on (1) total fatigue level 6 and 9 months after randomization and measure the mental fatigue, physical fatigue, HRQoL, vitality, life satisfaction, symptoms of depression and anxiety, work-life, exercise competence, nutrition, and physical fitness 3, 6, and 9 months after randomization; (2) HRQoL among partners 3, 6, and 9 months after randomization; (3) cost-utility of the intervention; and (4) the participants’ experiences (eg, perceived benefits and use of strategies in daily life) from participating in the intervention as a whole and from the individual components of the program along with measuring the levels of fatigue, HRQoL, depressive and anxiety symptoms, and work-life within each group at the 1- and 2-year follow-ups.

## Methods

### Study Design and Participants

The REFUEL trial is a 2-armed RCT comparing the effect of a 12-week multidisciplinary intervention to usual care among lymphoma survivors with CF.

Participants in both groups undergo assessments before randomization (T0), 3 months after randomization (T1), 6 months after randomization (T2), and 9 months after randomization (T3). Randomization ends after T3 (the usual care group is offered a modified intervention). Selected patient-reported outcomes are assessed in the total sample at the 1- (T4) and 2-year (T5) follow-up.

Survivors diagnosed with Hodgkin lymphoma or aggressive non-Hodgkin lymphoma between 2010 and 2020; who had completed treatment with curative intent at Oslo University Hospital (OUS) or St Olavs Hospital, Trondheim University Hospital more than 2 years before inclusion; who were aged between 18 and 70 years at the time of inclusion; and meet the criteria for CF according to Chalder Fatigue Questionnaire (FQ) are eligible [32]. Additional inclusion and exclusion criteria are listed in Textbox 1.

**Inclusion and exclusion criteria for participation in the Randomized Controlled Trial in Chronically Fatigued Lymphoma Survivors trial.**

**Inclusion criteria**

- Survivors of Hodgkin or aggressive non-Hodgkin lymphoma
- Diagnosed between 2010 and 2020
- Received treatment with curative intent
- ≥2 years since treatment completion
- Aged between 18 and 70 years at inclusion
- Chronic fatigue measured by the Chalder Fatigue Questionnaire
- Able to understand the Norwegian language
- Participation approval from an oncologist (assessed during the medical screening)

**Exclusion criteria**

- Diagnosis of central nervous system lymphoma
- Ongoing cancer treatment
- Second cancer
- Persisting fatigue >1 year before the cancer diagnosis
- Somatic or physical conditions (ie, severe heart failure or disease, lung disease, and use of wheelchair or crutches)
- Psychiatric or mental disorders (ie, dementia, severe depression, and schizophrenia; assessed during the medical screening)
- Use of stimulants for attention-deficit/hyperactivity disorder
- Substance abuse disorder

### Recruitment

Potential eligible participants are identified through the Lymphoma Register at OUS or the treatment database Cytodose at St Olavs Hospital, and receive written study information, consent form, and Chalder FQ by mail. In the invitation letter, those who have experienced persistent tiredness or exhaustion and wish to participate are asked to fill out the FQ, sign the written consent form, and return this in a prepaid response envelope. Survivors who do not respond to the invitation within 3 to 4 weeks receive a reminder via an SMS text message. The study coordinator contacts all respondents by phone to provide additional details concerning study participation. Participants who do not meet the criteria for CF will be excluded after this initial screening.

### Medical Screening

Eligible consenting survivors undergo a digital medical screening with an oncologist. The medical screening includes a detailed medical history related to lymphoma treatment and fatigue, evaluation of somatic and psychological comorbidities, and review of medications. Blood samples are obtained before the screening to assess hematological status; electrolytes; liver, biliary, and kidney functions; hormones; metabolism; and nutritional deficiencies. Details about the medical screening and blood samples are given in Multimedia Appendix 1.

If the medical screening and blood samples reveal or indicate conditions that make it unsafe or difficult to carry out the physical tests or exercise program and require further medical follow-up, the survivor and their general practitioner are informed. Findings from survivors with symptoms indicative of heart disease are discussed with a cardiologist (KHT), and the survivors are referred for further examination if indicated before inclusion or exclusion. Survivors passing the medical screening are referred for baseline assessments before randomization.

### Outcomes and Assessments

**Figure 1:**
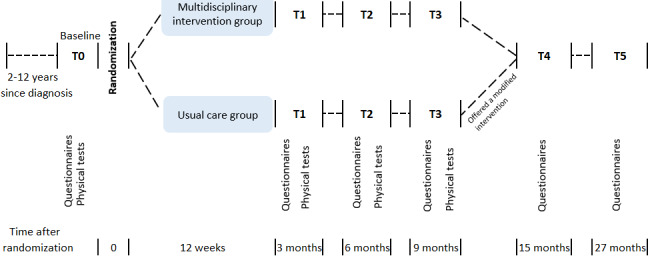
Study design. Participants in both groups undergo assessments before randomization (T0), at 3 months after randomization (T1), 6 months after randomization (T2), and 9 months after randomization (T3). Randomization ends after T3 (usual care group is offered a modified intervention). Selected patient-reported outcomes are assessed in the total sample at 1-year (T4) and 2-year (T5) follow-ups.

All outcomes are assessed at T0, T1, T2, and T3 (Figure 1). Selected patient-reported outcome measures (PROMS) and experience measures are assessed at T4 and T5 (15 and 27 months after randomization) in the whole sample. Outcomes, assessments, and time points of assessments are summarized in Table 1.

**Table 1:** Outcomes, assessments, and time points of assessments in the Randomized Controlled Trial in Chronically Fatigued Lymphoma Survivors trial.

Outcome (assessment)				Time point of assessment					
				T0^a^	T1^b^	T2^c^	T3^d^	T4^e^	T5^f^
**Among survivors**									
	**Primary outcome**								
		Level of fatigue (Chalder FQ^g^ [32])		✓	✓	✓^h^	✓^h^	✓^h^	✓^h^
	**Secondary outcomes**								
		**Questionnaires**							
			HRQoL^i^ (EORTC QLQ-C30^j^ [33])	✓	✓	✓	✓	✓	✓
			HRQoL and health status (EQ-5D [34])	✓	✓	✓	✓		
			Subjective vitality (SVS^k^ [35])	✓	✓	✓	✓		
			Satisfaction with life (SWLS^l^ [36])	✓	✓	✓	✓		
			Symptoms of depression (PHQ-9^m^ [37])	✓	✓	✓	✓	✓	✓
			Symptoms of anxiety (GAD-7^n^ [38])	✓	✓	✓	✓	✓	✓
			Work status (HUNT^o^ [39])	✓	✓	✓	✓	✓	✓
			Work ability (WAI^p^ [40])	✓	✓	✓	✓	✓	✓
			Perceived exercise competence (PCS^q^ [41])	✓	✓	✓	✓	✓	✓
			Diet (DIGIKOST-FFQ^r^ [42-45])	✓	✓	✓	✓		
		**Physical tests**							
			Cardiorespiratory fitness and pulmonary function (CPET^s^ or indirect cardiorespiratory fitness test^t^)	✓	✓	✓^u^	✓^u^		
			Leg strength (1 RM^v^ in leg press)	✓	✓	✓	✓		
			Upper body strength (maximum number of push-ups performed in 1 set)	✓	✓	✓	✓		
	**Additional measures (patient-reported experience measures)**								
		Perceived benefits of being part of the project (questions modified from the PasOpp survey^w^ [46])						✓	✓
		Perceived benefits of the 4 components of the intervention (self-made questions^w^)						✓	✓
		Use of experiences, strategies, and advice learned in the program (self-made questions^w^)						✓	✓
**Among partners**									
	HRQoL (RAND-36^x^ [47])			✓	✓	✓	✓		
	Global health or QoL (2 items; EORTC QLQ-C30 [33])			✓	✓	✓	✓		

^a^T0: before randomization.

^b^T1: 3 months after randomization.

^c^T2: 6 months after randomization.

^d^T3: 9 months after randomization.

^e^T4: 1-year follow-up (15 months after randomization).

^f^T5: 2-year follow-up (27 months after randomization).

^g^FQ: Fatigue Questionnaire.

^h^Fatigue levels at T2, T3, T4, and T5 are secondary outcomes.

^i^HRQoL: health-related quality of life.

^j^EORTC QLQ-C30: European Organization for Research and Treatment of Cancer Core Quality of Life Questionnaire.

^k^SVS: Subjective Vitality Scale.

^l^SWLS: Satisfaction With Life Scale.

^m^PHQ-9: Patient Health Questionnaire-9.

^n^GAD-7: Generalized Anxiety Disorder-7.

^o^HUNT: Trøndelag Health Study.

^p^WAI: Work Ability Index.

^q^PCS: Perceived Competence Scale.

^r^DIGIKOST-FFQ: digital food frequency questionnaire.

^s^CPET: cardiopulmonary exercise test.

^t^Participants not able to travel to Oslo University Hospital for CPET are offered an alternative indirect treadmill test, administered by an instructor at a local training facility.

^u^Pulmonary function is not assessed at T4 and T5.

^v^RM: repetition maximum.

^w^Refer to Multimedia Appendix 2.

^x^RAND-36: Research and Development 36-item Short Form Health Survey.

### Primary Outcome

Fatigue is measured by the Chalder FQ [32]. The FQ consists of 11 items, including a physical (7 items) and a mental (4 items) fatigue scale. Each item has 4 response categories scored from 0 to 3. The sum of the physical fatigue score (0-21) and mental fatigue score (0-12) provides the total fatigue score (0-33). Higher scores imply more fatigue. To identify cases with CF, scores of all 11 items (0-3) are dichotomized (0=0, 1=0, 2=1, and 3=1) [32]. CF is defined by a sum score ≥4 and fatigue duration for 6 months or more.

### Secondary Outcomes

HRQoL is assessed by the European Organization for Research and Treatment of Cancer Core Quality of Life Questionnaire [33]. This questionnaire includes 5 scales that measure function (physical, role, cognitive, emotional, and social function), 3 scales that measure symptoms (fatigue, pain, and nausea or vomiting), 6 scales that measure single items (dyspnea, insomnia, appetite loss, constipation, diarrhea, and financial difficulties) and a scale that measures the global health status and QoL. Items in the scales that measure function and symptoms are rated from 1 (not at all) to 4 (very much), while the global health status and QoL items are rated from 1 (very poor) to 7 (excellent). All scales and items are transformed to 0 to 100 scales according to the EORTC scoring manual. Increasing scores on functioning scales and global health status and QoL imply better functioning and QoL, while increasing scores on symptom scales and single items imply more severe symptoms [33].

Health status and HRQoL are measured by EQ-5D-5L [34]. EQ-5D-5L consists of the 5 dimensions: mobility, self-care, usual activities, pain and discomfort, and anxiety and depression. Each dimension has 5 severity levels that are described by statements appropriate to that dimension, coded from 1 (no problems) to 5 (unable to perform activities or extreme level of symptoms). The participants also rate their health status from 0 (the worst health you can imagine) to 100 (the best health you can imagine). The participants’ health status and HRQoL measured by EQ-5D-5L will be used to assess the cost-utility of the intervention.

Life satisfaction is measured by the Satisfaction With Life Scale [35]. The scale consists of 5 items with statements regarding life satisfaction, scored from 1 (totally disagree) to 7 (totally agree), providing a sum score ranging from 5 to 35. An increasing score implies higher satisfaction with life.

Subjective vitality is measured by the 5-item version of the Subjective Vitality Scale [36]. The scale consists of 5 items that measure the level of energy and vitality, scored from 1 (totally disagree) to 7 (totally agree), with a sum score ranging from 5 to 35. Increasing scores reflect higher subjective vitality.

Symptoms of depression are measured by the Patient Health Questionnaire-9 [37], and symptoms of anxiety by the Generalized Anxiety Disorder 7-items [38]. Patient Health Questionnaire-9 consists of 9 items scored from 0 to 3, providing a total score from 0 to 27. Generalized Anxiety Disorder 7-items consists of 7 items scored from 0 to 3, providing a total score from 0 to 21. Items on both scales are consistent with the *Diagnostic and Statistical Manual of Mental Disorders, Fifth Edition* diagnostic criteria for depression and anxiety, respectively. Higher scores imply a higher level of depressive and anxiety symptoms.

Work status is assessed by questions regarding current work situation, using the same questions as in the Trøndelag Health Study (HUNT) [39]. Response options include: full-time work, part-time work, job-seeking, laid off, sick leave, work assessment allowance, disability pension, self-employed, retired, military service, education, homemaker, and other. Additional questions address the duration and degree of sick leave and disability pension.

Work ability is evaluated using 3 items from the original 7-item Work Ability Index [40]. Physical and psychological work ability in relation to the demands of the job are each rated by the following response alternatives: very good, rather good, moderate, rather poor, or very poor. Current work ability is assessed by the Work Ability Score, which includes current overall work ability compared to the participant’s lifetime best work ability on a scale from 0 (extinguished work ability) to 10 (highest possible work ability).

Perceived exercise competence is assessed with an exercise version of the 4-item Perceived Competence Scale [41]. The participants respond to 4 statements related to their ability to be physically active on a 7-point Likert scale ranging from 1 (not at all true) to 7 (very true). A higher average sum of the 4 items reflects higher perceived exercise competence.

Diet is assessed by the digital DIGIKOST–food frequency questionnaire (FFQ) [42-45]. DIGIKOST-FFQ is a digital diet and lifestyle assessment tool designed to measure adherence to the Norwegian food-based dietary guidelines [48]. The questionnaire covers intake (in grams per day) of the main food groups reported in the last 2 weeks, including foods rich in fiber (ie, fruits, berries, vegetables, and whole grain products), fish (fatty and lean fish), unsalted nuts, dairy products (high- and low-fat content), meat (red, white, and processed meat), oils, margarine, foods rich in sugars and fat, sugar-rich beverages, and dietary supplements. DIGIKOST-FFQ also includes the Norwegian diet index and the Norwegian lifestyle index. The Norwegian diet index consists of 12 components corresponding to the Norwegian food-based dietary guidelines with a 3-level scoring approach, including 3 categories representing low, intermediate, and high adherence, giving a composite diet index ranging in scores from 0 (lowest adherence) to 20 (highest adherence). The Norwegian lifestyle index consists of 5 components (ie, diet, physical activity, normal weight, alcoholic drinks, and tobacco use) with a 3-level equal scoring approach and a composite lifestyle index ranging from 0 to 5 points [45]. Participants receive a link to DIGIKOST-FFQ via an SMS text message at the same time points as the other assessments.

Cardiorespiratory fitness and pulmonary function are assessed by a cardiopulmonary exercise test (CPET) conducted at OUS. Cardiorespiratory fitness, expressed as peak oxygen consumption (VO_2peak_), is assessed using a modified Balke treadmill protocol [49,50]. Before the test, participants are briefly familiarized with treadmill walking. The protocol starts at a speed of 4.8 kilometers per hour and with a 4% incline. The incline increases by 2% per minute until reaching a 20% incline, after which the speed increases by 0.5 kilometer per hour each minute. The test continues until the participant reaches voluntary exhaustion. Expired gases are measured continuously, breath-by-breath, using a gas and volume calibrated metabolic chart (SentrySuite, CareFusion, Jaeger). The highest volume of oxygen consumed during a 30-second period is recorded as VO_2peak_. Pulmonary function is assessed before the treadmill protocol at T0 and T1 by spirometry or flow-volume for pulmonary function (forced vital capacity, forced expiratory volume in the first second, maximal voluntary ventilation, and diffusion capacity for carbon monoxide in the lung).

If participants are unable to travel to Oslo for the CPET, they undergo the same modified Balke protocol at their local training center, continued until voluntary exhaustion as in CPET [49,50]. This indirect test is led by a physiotherapist or exercise physiologist in the municipality. At completion, the instructor registers time to exhaustion, heart rate (HR)_peak_, speed, and incline. Test results (eg, maximum speed and incline, as well as age and body weight) are then applied in a VO_2peak_ prediction equation.

Muscle strength is assessed by 1-repetition maximum test, that is, the maximal workload that can be lifted once in the leg press. For upper body strength, maximum number of push-ups performed in 1 set is registered.

### Additional Measurements

Perceived benefits of taking part in the project are assessed by questions modified from the PasOpp survey, developed by the Norwegian Institute of Public Health to measure patient experiences with rehabilitation institutions [46]. Use of experiences, strategies, and advice from the program in daily life is assessed by self-made questions (Multimedia Appendix 2).

HRQoL among the partners is measured by the Research and Development 36-item Short Form Health Survey (RAND-36) [47] and the global health status or QoL scale from the EORTC QLQ C-30 (described earlier) [33]. RAND-36 consists of 36 items assessing 8 health concepts: physical functioning (10 items), role limitations caused by physical health problems (4 items), role limitations caused by emotional problems (3 items), social functioning (2 items), emotional well-being (5 items), energy and fatigue (4 items), pain (2 items), and general health perceptions (5 items). In addition, 1 item assesses current perceived health status compared to the perceived health status 1 year ago. Physical and mental health summary scores are also derived from the 8 RAND-36 scales. Scale scores are summed and then transformed to scales ranging from 0 (worst possible health state) to 100 (best possible health state) [47].

### Background Variables

Sociodemographic variables include age, gender, education level, and marital status, and are assessed by questions used in the HUNT study [39].

The work environment is measured by the Demand Control Support Questionnaire [51]. It includes 17 statements concerning psychological demands (5 items), decision latitude (6 items), and social support (6 items) at the workplace. The level of agreement with these statements is reported on a 4-point scale ranging from 1 (complete agreement) to 4 (no agreement at all). Higher scores indicate higher psychological demands (range 5-20), higher decision latitude (range 6-24), and higher social support at work (range 6-24).

Cancer-related variables include information on cancer diagnosis and treatment, and comorbidities extracted from the medical record and the medical screening.

Physical activity level is measured by a modified version of the Godin Leisure-Time Exercise Questionnaire [52]. It assesses the average frequency and duration of mild, moderate, and vigorous leisure-time physical activity during a typical week. By summing the weekly minutes of moderate and vigorous intensity levels of physical activity, participants are dichotomized into meeting or not meeting the World Health Organization guidelines on physical activity (ie, 150-300 min of moderate intensity or 75-150 min of vigorous intensity physical activity per week, or an equivalent combination of moderate and vigorous intensity activity throughout the week [53]).

BMI is calculated from height and weight (kg/m^2^), measured at the physical tests.

Smoking, snuff, and alcohol consumption are measured by questions used in the HUNT study [39].

Sleep problems are assessed by the following question extracted from the HUNT study [39]: “How often in the last 3 months have you experienced difficulty falling asleep, waking up repeatedly through the night, woken too early and could not get back to sleep, and difficulty coping during the daytime (socially or professionally) due to sleep problems?” Response alternatives are “never,” “seldom,” “sometimes” and “3 times a week at least.”

Personality is measured by a short version of the Big Five Inventory [54]. It measures the 5 personality domains: openness, conscientiousness, extraversion, agreeableness, and neuroticism. The questionnaire consists of 20 items, distributed into 4 items for each personality domain. Items are scored on a Likert scale from 1 (the item does not describe the respondent at all) to 7 (the item describes the respondent very well). Scores range from 4 to 28 for each personality domain.

### Randomization and Blinding

After completion of baseline assessments, participants are allocated in a 1:1 ratio between the intervention group and usual care group using a block randomization procedure with random block sizes of n=2 and n=4. A computer-generated randomization list is generated using Stata statistical software (version 17.0; Stata Corporation). The allocation sequence is prepared by an independent statistician.

Details of block size and allocation sequence generation are unavailable to those who enroll patients or assign study group. Participants and assessors involved in baseline assessments are blinded to group assignment until baseline assessments are completed. Assessors are not blinded to group assignment at follow-up assessments. Due to the nature of the intervention, participants, physiotherapists, and health personnel delivering the intervention are not blinded to group assignment after randomization.

### Intervention

#### Overview

The multidisciplinary intervention is organized as shown in Table 2.

**Table 2:** Organization of the intervention components in the Randomized Controlled Trial in Chronically Fatigued Lymphoma Survivors trial.

	Intervention period (week)											
	1	2	3	4	5	6	7	8	9	10	11	12
Patient education session	✓											
Physical exercise program	✓	✓	✓	✓	✓	✓	✓	✓	✓	✓	✓	✓
CBT^a^-based group program			✓	✓	✓	✓	✓	✓				
Individual nutritional counseling		✓				✓				✓		

^a^CBT: cognitive behavioral therapy.

#### Patient Education

The intervention begins with a 2-hour digital group–based patient education during the first week. Health care professionals from the Cancer Rehabilitation Centre at OUS provide information about CF (by an oncologist) and physical exercise and recovery (by a physiotherapist); an introduction to the CBT-based group program (by a psychologist); and information about diet and nutrition (by a registered clinical dietitian), all topics related to CF. The participants are encouraged to share experiences and ask questions during and at the end of the session.

#### Physical Exercise Program

The physical exercise program begins in the first week and continues throughout the whole intervention period. The program consists of 2 aerobic and strength training sessions per week, 1 supervised by a physiotherapist in the municipality and 1 unsupervised. The physiotherapists ensure that the exercise sessions are performed as planned and adjust the sessions if necessary. In the unsupervised sessions, the participants are instructed to follow the same program as in the supervised sessions. Each session lasts 30 to 60 minutes. An overview of the exercise program is provided in Tables 3 and 4.

**Table 3:** Overview of the aerobic exercise program in the Randomized Controlled Trial in Chronically Fatigued Lymphoma Survivors trial.

Aerobic exercise	Weeks 1 and 2 (familiarization)	Weeks 3 to 6	Weeks 7 to 12
Peak HR^a^ (%)	60-75	75-85	80-90
Subjective experience; Borg scale (6-20)	Light; 8-10	Light to moderate; 11-13	Moderate to hard; 12-15
Total duration of intervals (min)	8-12	8-12	8-12

^a^HR: heart rate.

**Table 4:** Overview of the strength exercise program in the Randomized Controlled Trial in Chronically Fatigued Lymphoma Survivors trial.

Strength exercise	Weeks 1 and 2 (familiarization)	Weeks 3 and 4	Weeks 5 to 12
Reps × sets per exercise	8 to 12 repetitions; 1 to 2 sets per exercise	8 to 12 RM^a^; 1 to 2 sets per exercise^b^	8 to 12 RM; 2 to 3 sets per exercise^c^
Subjective experience; RPE^d^ (1-10)	Light, focus on correct technique	Hard; 8-10	Hard; 8-10

^a^RM: repetition maximum, the load that induces technique failure in 8 to 12 repetitions.

^b^Week 1 to 4: push-ups, rowing, and dead bugs are performed in 1 set, and squats in 2 sets.

^c^Week 5 to 12: push-ups, rowing, and dead bugs are performed in 2 sets and squats in 3 sets.

^d^RPE: rate of perceived exertion.

The aerobic exercise component begins with 10 to 15 minutes of warm-up at low intensity, followed by intervals of moderate to high intensity exercise (Multimedia Appendix 3). The number and duration of intervals are flexible, as long as the total duration of the intervals is between 8 and 12 minutes. The mode most often used is uphill walking, outside or on a treadmill. Resting periods between intervals are at least half the duration of each interval.

The participants wear an HR monitor (Polar Verity Sense) to track their HR during the aerobic exercise. The intensity of the intervals is individually tailored to each participant based on their CPET or indirect VO_2peak_ test result at baseline. In the first 2 weeks, light-intensity intervals (60%-75% of peak HR) are conducted to familiarize the participant with the program. Throughout the program, the intensity of the intervals progressively increases (Table 3).

The strength training includes 4 mandatory exercises (variants of push-ups, dead bugs, standing rowing, and squats), along with 2 optional exercises (biceps curl and shoulder press, and variants of hip thrust). Each exercise is performed for 8 to 12 repetitions across 1 to 3 sets (Table 4 and Multimedia Appendix 3). Once a participant can complete 12 repetitions with proper technique, the physiotherapist increases the load (eg, using elastic bands with more resistance or heavier dumbbells). Alternatively, the physiotherapist introduces a more challenging exercise variation targeting the same muscle group (eg, from bodyweight squats to split squats). During the first 2 weeks, the focus is on proper technique. Through the following weeks, the physiotherapist gradually increases the load, difficulty, and number of sets.

After the initial 12-week exercise program, the physiotherapist and participant are encouraged to prepare an individual exercise plan for the next 12 weeks tailored to the participant’s exercise preferences and motivation. The physiotherapists are also encouraged to provide a follow-up call every second to fourth week to support exercise adherence.

With assistance from their physiotherapist, the participants record their sessions in an exercise log (Multimedia Appendix 4). For aerobic exercises, participants log the total number of intervals, the duration of each interval, their HR at the end of each interval, and their subjective experience of the aerobic session using the Borg Rating of Perceived Exertion scale (range 6-20). For strength exercises, participants log the number of repetitions, sets, and exercise variants for each completed exercise. Their subjective experience is recorded with an Rating of Perceived Exertion Vas Scale (range 1-10).

In the exercise log, participants also rate their level of fatigue the day after exercise on a scale from 1 (no fatigue) to 10 (maximum fatigue). If necessary, the physiotherapists adjust the training intensity and load according to the participants’ subjective experience. If a session results in significant fatigue that persists for the following days after the session, the participant is advised to reduce the intensity and duration of the subsequent session.

If the participant’s motivation and compliance diminish, or if injuries and physical complaints prevent the participant from performing the prescribed exercise, the physiotherapists make necessary individual adjustments (eg, alternative exercise modes, such as ergometer bike, rowing machine, and cross-country skiing) to prevent dropout. Any individual modifications to the program are registered in the exercise log.

#### CBT-Based Group Program

The CBT-based program starts in week 3. The program consists of 6 weekly digital group-based sessions, each led by 2 clinical psychologists (6-10 participants per group). Each session lasts for 2 hours and 15 minutes. The program is a modified version of a course designed to target fatigue in individuals with inflammatory rheumatic disease, developed by clinical psychologists at Diakonhjemmet Hospital, Oslo [55]. The aim of the program is to address behavioral and cognitive factors related to the participant’s symptoms and disability, especially strengthening perceived self-efficacy in the management of fatigue. The program incorporates key elements from CBT, including the following:

- Education about the biopsychosocial model of fatigue and teaching the participants to use the 5 areas model [56] to investigate the impact of bodily reactions, thoughts, feelings, behavior, and environmental factors in everyday situations.
- Making an activity plan for activity and rest, a tool for changing unhelpful behavior, and reducing the selective attention to fatigue.
- Guiding participants to address unhelpful cognitions, such as self-criticism and fears related to symptoms and activity, for example, by using reflective questions.
- Using mental strategies for unhelpful and excessive worry and rumination.
- Using implementation intentions and strategies for change of habits.
- Addressing social and emotional barriers to improvement through problem-solving.

Between group sessions, the participants spend about 45 to 60 minutes per week on homework and practice.

#### Nutritional Counseling

The nutritional counseling consists of 3 individual digital sessions in weeks 2, 6, and 10. Each session lasts for 30 to 60 minutes, all led by a registered clinical dietitian. The sessions aim to improve adherence to the Norwegian food-based dietary guidelines, address specific nutritional needs, and establish healthy meal patterns [57]. DIGIKOST-FFQ is used to estimate adherence to the Norwegian food-based dietary guidelines at baseline [42-45]. This assessment provides a digital benchmark of the individual’s diet and nutritional status, forming the basis for the counseling (ie, the DIGIKOST-report). Each consultation uses motivational interviewing techniques to support each individual participant in setting 1 to 3 dietary goals. The registered clinical dietitian encourages the survivor in achieving these goals, which are reviewed and revised at subsequent sessions [58].

#### Tolerability and Adherence to the Intervention

Tolerability of the intervention is assessed as loss to follow-up, and adherence to the separate intervention components and the intervention as a whole.

Adherence to the patient education, CBT sessions, and nutrition counseling is assessed by calculating the attendance rate for each of the components (dividing the number of attended sessions by the number of planned sessions).

Adherence to the exercise program is assessed based on information in the exercise log registrations. The attendance rate will be calculated by dividing the number of sessions registered in the exercise log by the planned number of sessions. The total number of interval sessions completed at the planned intensity, above or below the planned intensity (% of peak HR) is also reported.

For each part of the intervention, a participant is considered adherent if attending at least 65% of the offered sessions (ie, exercise sessions: 16/23, 70%; CBT-based group sessions: 4/6, 67%; and nutritional counseling sessions: 2/3, 67%). For the total intervention, a participant is defined as adherent if they have attended the patient education and at least 67% of each of the other parts of the intervention (ie, the combination of attending the patient education, ≥16 exercise sessions, ≥4 CBT sessions, and ≥2 nutritional counseling sessions).

A participant is considered adherent to usual care if their VO_2peak_ or self-reported physical activity level increased by less than 2 times the SD of the mean change in the intervention group.

#### Safety

All participants undergo an extensive medical screening before inclusion (described earlier) to ensure that it is safe for them to perform the exercise protocol.

Participants in the intervention group are frequently in contact with the study personnel, health professionals at OUS, and the physiotherapist in the municipality throughout the intervention. The physiotherapist monitors and assesses fatigue and activity levels, discussing this continuously with the participants. If the participants feel that the program is too extensive, it is individually adjusted as needed in collaboration with the study personnel.

Both during physical tests and the intervention, any adverse events that occur are registered by the participant, test personnel, or physiotherapist. If indicated, the incidence and severity of adverse events (eg, adverse events requiring medical treatment, causing persistent worsening of the participant’s health, requiring restrictions or early termination of the planned testing or training, or resulting in persistent or significant disability) are discussed with the study coordinator (SKHB and GMG), the principal investigator (LT), the medical responsible physicians (M Seland and AF) or the steering group (LT, CEK, and TS).

### Usual Care

The usual care group is encouraged to continue their lives as usual, and to maintain the same level of physical activity as at the time of inclusion. Nine months after randomization (after T3), participants in the usual care group are offered a modified version of the intervention in line with their personally expressed needs.

### Statistical Power and Analyses

Power calculation for the primary outcome, that is, total fatigue measured by the FQ was based on results from the pilot study by Oldervoll et al [59] and results from our feasibility study [31]. To detect a mean difference of 3.3 points (SD 6.1) in total FQ score between groups at T1 (no change in the usual care group and 3.3 points reduction in the intervention group) with a 2-sided significance level of 5% and a power of 80%, 54 survivors are required in each group. To have a sufficient sample size to detect a 10% group difference in the secondary HRQoL outcomes with similar assumptions, 62 survivors are needed in each group. Assuming a 20% loss to follow-up, 74 survivors have to be recruited in each group.

For the descriptive analyses, continuous variables will be presented as means and SDs or medians and ranges, while categorical variables will be presented by numbers and percentages.

To analyze the difference in total fatigue at T1 between groups, we plan to use a generalized linear mixed model, adjusting for baseline fatigue score and random effects for hospital. To analyze between-group differences in secondary outcomes at T1, T2, and T3, we will use similar analyses. If the assumptions for the generalized linear mixed model are not met, we will use alternative analyses. A *P* value <.05 will be considered statistically significant. The main analysis will be according to the intention-to-treat principle, including all randomized participants.

Per-protocol analyses will be performed by including only participants adherent to the randomized group.

To explore if the intervention effect on primary and secondary outcomes differs across subsets of participants (eg, age groups, gender, and time since diagnosis), we will also perform subgroup analyses.

Analyses of cost-utility will be performed based on data on resource consumption, HRQoL, and survival during the trial. The health outcome will be based on a combination of EQ-5D-5L [34] and patient survival, that is, the area under the curve, resulting in quality-adjusted life expectancy. Costs for the intervention will be calculated in detail using microcosting. Costs during follow-up will be based on recorded resource consumption and costed according to national and international guidelines for health economic evaluations and health technology assessments [60].

More details on the statistical analyses are provided in the statistical analysis plan Multimedia Appendix 5.

### Ethical Considerations

The trial is preregistered on ClinicalTrials.gov (NCT05130099). Protocol modifications are continuously registered on ClinicalTrials.gov. This protocol paper is written according to the SPIRIT (Standard Protocol Items: Recommendations for Interventional Trials) guidelines (Multimedia Appendices 6 and 7) [61].

#### Human Subject Ethics Review Approvals

The REFUEL trial is approved by the Regional Committees for Medical and Health Research Ethics (153665), the Data Protection Officer at OUS (20/19374) and the Research Committee of the Department of Oncology at St Olavs Hospital (04_2022_04). All study procedures are conducted in accordance with the Helsinki declaration.

#### Informed Consent

All study participants provide signed informed consent before inclusion in the study. The participation is voluntary, and participants can withdraw from the study at any time without giving any reason.

#### Privacy and Confidentiality

All study-related information will be stored securely at the study sites (OUS and St Olavs Hospital). All participant data will be identified by a coded identification number only to maintain participant confidentiality. Listings that link participant identification numbers to other identifying information will be stored in a separate locked file in an area with limited access. No identifying information or features of the participants will be published.

#### Compensation Details

Participants were not offered any incentives or compensation.

## Results

The project was granted funding by the Dam Foundation in October 2020. The first participant was included in November 2021 (Multimedia Appendix 8). Inclusion of 150 participants was completed in March 2023, of which 75 participants were randomized to the intervention and 75 participants to usual care. All T1 assessments were completed in June 2023 and the 1-year follow-up (T4) was completed in June 2024. The 2-year follow-up (T5) was completed in June 2025.

## Discussion

### Anticipated Findings

Recent fatigue guidelines from the American Society of Clinical Oncology highlight the lack of RCTs, including fatigued cancer survivors [1]. As the first RCT to test the effects of a multidisciplinary intervention on fatigue levels, HRQoL, and physical fitness in lymphoma survivors with CF, the results from the REFUEL trial will be an important contribution to this evidence gap. We hypothesize that survivors randomized to the multidisciplinary intervention will improve their level of total fatigue (primary outcome). Moreover, we hypothesize that the intervention will be effective on other HRQoL outcomes and physical fitness from before to immediately after the intervention, compared to survivors randomized to usual care.

### Comparison to Previous Work

Exercise and psychological interventions are recommended to improve fatigue among cancer survivors during and after treatment [1,19,27]. However, the effects are small to moderate, and the existing evidence base has several limitations [1]. There is a lack of studies with fatigue as the primary outcome, and very few studies have screened the participants for fatigue before inclusion, particularly in long-term cancer survivors [1]. By including lymphoma survivors with CF according to well-established criteria, this RCT represents a significant contribution to the existing evidence base.

To date, the few studies that have combined multiple components to address fatigue after cancer have yielded inconsistent results. A meta-analysis showed that the combination of exercise and psychological interventions were sometimes equivalent to or inferior to a single modality [19]. Combined interventions could be counterproductive if the content is insufficient or if the intervention places too high a burden on participants or is too time-consuming, leading to reduced adherence and high dropout rates [19]. On the other hand, given the complex mechanisms underlying CF, it is plausible that integrating education, physical exercise, psychological strategies, and nutrition could have synergistic effects, leading to larger fatigue reductions compared to single modality interventions. The REFUEL trial aims to provide a more definitive conclusion regarding the effects of combined interventions.

The REFUEL intervention was developed through close collaboration between clinicians and researchers with extensive experience in CF among cancer survivors. However, we acknowledge that the intervention is ambitious and demands considerable efforts from the participants, study personnel, and health professionals delivering the intervention. As a preparation for the RCT, we tested the intervention in a feasibility study [31]. In order to prevent worsening of fatigue and dropout among the participants, as well as to ensure that the study personnel and health professionals are well prepared, we learned that it is crucial to ensure sufficient time for information, planning, and coordination. We also made some adjustments to reduce the total load of the intervention, in accordance with feedback provided by the survivors testing the intervention in the feasibility study [31]. Finally, a close dialogue between the study personnel, health professionals, and the participants is important throughout the trial to detect and address challenges, and if necessary, make individual adjustments to the intervention.

### Strengths and Limitations

A key strength of this study is the multidisciplinary intervention targeting several modifiable factors associated with CF, developed through close collaboration between clinicians and researchers.

Another strength of this intervention is the integration of digital services with supervised exercises by local physiotherapists, ensuring that travel distance was not a barrier to study participation. Combining digital and home-based interventions can help mitigate disparities in geographical access to fatigue rehabilitation services and thereby promote equality in health care services.

Limitations of the study include the risk of recruiting the most motivated individuals, and the need to exclude those with medical conditions that are incompatible with the study, which will have to be taken into account when considering the external validity of our results.

Another limitation of this study is the lack of blinding of study personnel conducting the physical assessments. However, this is not likely to affect the primary outcome or the additional secondary outcomes, as these were evaluated directly by the participants through PROMS. We consider PROMS the most efficient and appropriate assessment method for subjective experiences that cannot be captured by objective markers or clinical assessments (eg, fatigue). Nevertheless, we acknowledge that these measures are prone to bias, such as response bias and responses influenced by fluctuations in mood and expectations of the participants, which can lead to inconsistencies in the data. Efforts to reduce the disadvantages associated with self-report have been made by using validated PROMS and combining PROMS with objective physical assessments.

Another concern is poor intervention adherence and dropouts. To prevent dropout from the intervention group, the exercise dose is individually tailored according to baseline test results. The exercise sessions are supervised by experienced physiotherapists who will adjust the program if needed. To prevent dropout in the usual care group, they are offered a modified version of the intervention after completion of the assessments, 9 months after randomization.

### Future Directions

The results from the REFUEL trial can inform future fatigue guidelines. If proven effective, this intervention provides support for a multidisciplinary approach in clinical practice to improve fatigue among cancer survivors.

## Appendix

Multimedia Appendix 1 Medical screening.

Multimedia Appendix 2 Patient-reported experience measures.

Multimedia Appendix 3 Physical exercise program instructions.

Multimedia Appendix 4 Example of exercise log.

Multimedia Appendix 5 Statistical analysis plan.

Multimedia Appendix 6 SPIRIT (Standard Protocol Items: Recommendations for Interventional Trials) checklist.

Multimedia Appendix 7 Time schedule of enrollment, intervention, and assessments in the Randomized Controlled Trial in Chronically Fatigued Lymphoma Survivors trial.

Multimedia Appendix 8 Peer-review report from Foundation DAM.

## Abbreviations

CBT: cognitive behavioral therapy
CF: chronic fatigue
CPET: cardiopulmonary exercise test
FQ: Fatigue Questionnaire
HR: heart rate
HRQoL: health-related quality of life
HUNT: Trøndelag Health Study
OUS: Oslo University Hospital
PROMS: patient-reported outcome measures
RAND-36: Research and Development 36-item Short Form Health Survey
RCT: randomized controlled trial
REFUEL: Randomized Controlled Trial in Chronically Fatigued Lymphoma Survivors
SPIRIT: Standard Protocol Items: Recommendations for Interventional Trials
VO_2peak_: peak oxygen consumption

## Data Availability

The data that will be generated and analyzed during this study is not publicly available due to privacy and ethical restrictions. The data or parts of the data are available from the corresponding author upon reasonable request.
