# Supplementary material for: Effects of a Multidisciplinary Intervention on Fatigue in Lymphoma Survivors With Chronic Fatigue: Protocol for a Randomized Controlled Trial (REFUEL)
Source: JMIR Res Protoc. 2025 Aug 29;14:e69336. doi: 10.2196/69336 (PMC12432467; doi:10.2196/69336)
Supplement: Multimedia Appendix 1 [file resprot_v14i1e69336_app1.pdf]

Medical screening	
**Fatigue history**	• Time of debut<br>• What triggered fatigue (cancer diagnosis? Other)<br>• Is the fatigue constant or intermittent?<br>• Consequences on daily life?<br>• Persisting fatigue > 1 year before cancer diagnosis? E.g. sick leave because of fatigue? ME diagnosis?
**Lymphoma history**	• Diagnosis and treatment<br>• Approximate time of and (patient reported) conclusion of last follow-up (at the hospital or GP)<br>• New symptoms or health problems since last follow-up at the hospital or GP? Suspected relapse?<br>• Current follow-up (at the hospital or GP) of late effects or conditions related to cancer or cancer treatment (e.g. lymphedema, heart disease or neuropathy)?<br>• Women: regular menstrual cycle/age at menopause
**Somatic comorbidity**	• Cardiac disease (chest pain, palpitations, dyspnea, edema, hypertension)<br>• Lung disease (severe asthma, chronic obstructive pulmonary disease)<br>• Diabetes<br>• Arthritis or other diseases/major musculoskeletal injury limiting daily physical functioning (e.g. fibromyalgia, ME, use of wheel chair/crutches)<br>• Severe/uncontrolled pain limiting daily functioning<br>• Other comorbidities (endocrine- or renal dysfunction, anemia, pain)
**Psychological comorbidity/ substance abuse**	• Mental disorders (severe anxiety, depression, dementia or other mental condition) that have resulted in follow-up from the specialist health care service<br>• Problems with drugs (alcoholism or substance abuse) that have resulted in follow-up from the specialist health care service
**Medications**	Regularly or when needed (including dietary supplements, hormone substitution, contraceptions)
**Blood samples**	• Hematologic status (Hb, erythrocytes, EVF, MCH, MCHC, MCV, trombocytes, leukocytes with differential count)<br>• Electrolytes (Na, K, Cl, Ca)<br>• Liver/biliary- and kidney tests (creatinine, ALAT, LD, ALP, albumin)<br>• Hormones/metabolism TSH, FT4, FSH, LH, SHBG, testosterone and estrogen<br>• Nutritional deficiencies (serum ferritin, Vitamin B9 (folate), Vitamin B_{12} (cobalamin), vitamin D (25-OH-vitamin D total)).
