# Supplementary material for: Effects of a Multidisciplinary Intervention on Fatigue in Lymphoma Survivors With Chronic Fatigue: Protocol for a Randomized Controlled Trial (REFUEL)
Source: JMIR Res Protoc. 2025 Aug 29;14:e69336. doi: 10.2196/69336 (PMC12432467; doi:10.2196/69336)
Supplement: Multimedia Appendix 2 [file resprot_v14i1e69336_app2.pdf]

YOUR EXPERIENCES WITH PARTICIPATING IN THE REFUEL-STUDY

1. To what extent have you benefitted from participating in terms of:

	No benefit	Small benefit	Some benefit	Large benefit	Very large benefit	Don't know/not applicable
Your physical health	☐	☐	☐	☐	☐	☐
Your mental health	☐	☐	☐	☐	☐	☐
Ability to cope with daily tasks	☐	☐	☐	☐	☐	☐
Work participation or education	☐	☐	☐	☐	☐	☐
Taking part in social activities	☐	☐	☐	☐	☐	☐

2. Overall, to what extent have you benefitted from participating in the study?

No benefit	Small benefit	Some benefit	Large benefit	Very large benefit	Don't know/not applicable
☐	☐	☐	☐	☐	☐

3. Overall, how satisfied or dissatisfied are you with the services you received through the study?

Very dissatisfied	Somewhat dissatisfied	Neither satisfied nor dissatisfied	Quite satisfied	Very satisfied	Don't know/not applicable
☐	☐	☐	☐	☐	☐

4. Through your participation in the REFUEL-study, have you:

	Yes	No
Gained increased knowledge and understanding of what fatigue is	☐	☐
Gained increased knowledge and understanding of how to cope with living with fatigue	☐	☐
Learned methods and coping strategies for participation in the workforce	☐	☐
None of the above	☐	☐

PERCEIVED BENEFITS FROM PARTICIPATING IN THE STUDY

5. To what extent have you benefitted from the various components of the program?

	No benefit	Small benefit	Some benefit	Large benefit	Very large benefit	Don't know/ not applicable
The digital patient education (first week)	☐	☐	☐	☐	☐	☐
Exercise supervised by physiotherapist	☐	☐	☐	☐	☐	☐
The digital group sessions with psychologist	☐	☐	☐	☐	☐	☐
The nutritional counseling with registered clinical dietitian	☐	☐	☐	☐	☐	☐

6. In your experience, to what extent did the various components of the program contribute to reducing levels of fatigue during and shortly after the program?

	Not at all	To a small extent	To some extent	To a large extent	To a very large extent	Don't know/not applicable
The digital patient education (first week)	☐	☐	☐	☐	☐	☐
Exercise supervised by physiotherapist	☐	☐	☐	☐	☐	☐
The digital group sessions with psychologist	☐	☐	☐	☐	☐	☐
The nutritional counseling with registered clinical dietitian	☐	☐	☐	☐	☐	☐

7. In your experience, to what extent did the various components of the program contribute to improving your overall situation?

	Not at all	To a small extent	To some extent	To a large extent	To a very large extent	Don't know/not applicable
The digital patient education (first week)	☐	☐	☐	☐	☐	☐
Exercise supervised by physiotherapist	☐	☐	☐	☐	☐	☐
The digital group sessions with psychologist	☐	☐	☐	☐	☐	☐
The nutritional counseling with registered clinical dietitian	☐	☐	☐	☐	☐	☐

8. To what extent are you using the information/experiences/strategies/advice you acquired from the various components <u>today?</u>

	Not at all	To a small extent	To some extent	To a large extent	To a very large extent	Don’t know/not applicable
The digital patient education (first week)	☐	☐	☐	☐	☐	☐
Exercise supervised by physiotherapist	☐	☐	☐	☐	☐	☐
The digital group sessions with psychologist	☐	☐	☐	☐	☐	☐
The nutritional counseling with registered clinical dietitian	☐	☐	☐	☐	☐	☐

9. To what extent do the information/experiences/strategies/advice you acquired from the various components contribute to reducing fatigue levels/keeping fatigue levels stable <u>today?</u>

	Not at all	To a small extent	To some extent	To a large extent	To a very large extent	Don’t know/not applicable
The digital patient education (first week)	☐	☐	☐	☐	☐	☐
Exercise supervised by physiotherapist	☐	☐	☐	☐	☐	☐
The digital group sessions with psychologist	☐	☐	☐	☐	☐	☐
The nutritional counseling with registered clinical dietitian	☐	☐	☐	☐	☐	☐
