# Supplementary material for: Effects of a Multidisciplinary Intervention on Fatigue in Lymphoma Survivors With Chronic Fatigue: Protocol for a Randomized Controlled Trial (REFUEL)
Source: JMIR Res Protoc. 2025 Aug 29;14:e69336. doi: 10.2196/69336 (PMC12432467; doi:10.2196/69336)
Supplement: Multimedia Appendix 3 [file resprot_v14i1e69336_app3.pdf]

Physical exercise program instructions

The exercise program and intensity level:

- Each week, the participant completes two exercise sessions; one supervised by physiotherapist and one unsupervised
- The exercise program can be modified according to individual considerations, such as motivation and physical fitness
- **It is important that the participant and physiotherapist discuss level of fatigue and adjust if the exercise intensity is too high. Please contact us if you have any questions.**

<u>**Aerobic exercise:**</u>

- Begin with 10 minutes warm-up at low intensity (50-60% of HR_{max})
- Complete 2-6 intervals each session, with a total duration of 8 to 12 minutes
    - The number and duration of intervals are optional. Examples:
        - 4 intervals of 2 minutes duration
        - 3 intervals of 3 minutes duration
        - First interval 2 minutes duration, second 3 minutes, third 4 minutes

- **The rest period** between each interval should be at least half the duration of each interval

- **Intensity**

    - Week 1 and 2: **familiarization** to the exercise program, with **intensity of 60-75% of HR_{max} (light intensity). It is important that these first sessions are performed with light intensity.** Keep the intervals few and short, and focus on exercise competence

    - Week 3-6: intensity increases to **75-85% of HR_{max} (corresponding to light/somewhat hard on Borg Scale)**

    - From week 7: intensity **80-90% of HR_{max} (corresponding to hard on Borg Scale)**

Strength exercise:

- **Familiarization week 1 and 2:** focus on learning the exercises with appropriate technique with light load

- From week 3: perform 8-12 repetitions of each exercise with the maximum load that do not induce technique failure (8-12 RM)

- When the participant can complete 12 repetitions with proper technique, increase the load, alternatively introduce a more challenging exercise variation targeting the same muscle group

- In week 1 to 4 upper body exercises are performed in one set, and lower body exercises are performed in two sets

- From week 5: upper body exercises are performed in two sets, and lower body exercises are performed in three sets

How to enter the exercise log

- Log exercise information during/right after each session

<u>AEROBIC INTERVALS</u>

- Heart rate is logged at the end of each interval
- Any modifications are registered in the commentary-field
- In case of an interrupted training session, register the reason and the number of completed minutes/exercises
- Enter an overall Borg Scale value (6-20) for the aerobic intervals
- Complete planned exercise only
- Based on the number of intervals and duration completed, enter the planned number of intervals and duration for the next session

<u>STRENGTH EXERCISE</u>

- Enter number of sets, repetitions and exercise variation
- After each set: enter Rate of Perceived Exertion (RPE) from 1-10 (1: very light, 10: max effort)
- Based on the completed number of repetitions and sets, enter the planned number of repetitions and sets for each exercise for the next session

If you have any questions or in the case of adverse events, contact principal investigator Lene Thorsen or study coordinator Synne Bøhn (phone number xxxx/synn@ous-hf.no)

Calculation of exercise intensity

Estimated maximum heart rate (HR_{max}) based on treadmill test (5-10 beats above peak heart rate)

_______________beats/min

Based on estimated HR_{max}, enter the participant's minimum and maximum heart rate during the aerobic interval sessions below by multiplying estimated HR_{max} with per cent of HR_{max}. Example: HR_{max} = 190, estimate 80 % of HR_{max}: 190 x 0.8 = 152)

Week 1 and 2: intensity 60-75 % of HR_{max} (beats per minute):

min________ max_______

Week 3-6: intensity 75-85 % of HR_{max} (beats per minute):

min________ max________

Week 7-12: intensity 80-90 % of HR_{max} (beats per minute):

min_________max_______

The table below displays the correlation between Borg scale and heart rate based on a HR_{max} of 190 beats/minute.

**% HR_{max}**	**HR**	**Level of exertion**	**Borg**
80-90 %	152-171	Hard. Difficult to hold a conversation	12-16
75-85 %	137-162	Light/somewhat hard. Can hold short conversations	11-14
60-75 %	114-136	No exertion. Able to hold a conversation	6-11

HR: heart rate
