# Supplementary material for: Effects of a Multidisciplinary Intervention on Fatigue in Lymphoma Survivors With Chronic Fatigue: Protocol for a Randomized Controlled Trial (REFUEL)
Source: JMIR Res Protoc. 2025 Aug 29;14:e69336. doi: 10.2196/69336 (PMC12432467; doi:10.2196/69336)
Supplement: Multimedia Appendix 4 [file resprot_v14i1e69336_app4.pdf]

ID | | | |

Date | | | | | | | | |

Session nr. | | **Week nr.** | |

Supervised exercise session
Aerobic intervals: heart rate 80-90 % of HR_{max}
Strength exercises: 3 sets on lower body, 2 sets on upper body

**Aerobic exercise**			
**Based on the previous session, the plan is to complete ___ intervals of ____minutes duration**			
	Planned duration (minutes)	Completed duration (minutes)	HR at completion of interval
Interval 1			
Rest			
Interval 2			
Rest			
Interval 3			
Rest			
Interval 4			
Rest			
Interval 5			
Rest			
Interval 6			
Rest			
Completed duration of intervals (minutes)			
Borgs Scale for the total interval session (6-20)			

Strength exercises

Squats

	Repetitions	Sets	Exercise nr	RPE
Planned				
Completed				
Completed				
Completed				

Push-ups

	Repetitions	Sets	Exercise nr	RPE
Planned				
Completed				
Completed				

Rowing

	Repetitions	Sets	Exercise nr	RPE
Planned				
Completed				
Completed				

Dead bug

	Repetitions	Sets	Exercise nr	RPE
Planned				
Completed				
Completed				

Voluntary exercises:

Hip-thrust

	Repetitions	Sets	Exercise nr	RPE:
Planned				
Completed				
Completed				
Completed				

Biceps curls and shoulder press

	Repetitions	Sets	Exercise nr	RPE:
Planned				
Completed				
Completed				

The day after exercise: rate the level of fatigue on a scale from 0 (no fatigue) to 10 (maximum fatigue)	

Comments (e.g. modifications of the exercise plan, adverse events)
