# Supplementary material for: Effects of a Multidisciplinary Intervention on Fatigue in Lymphoma Survivors With Chronic Fatigue: Protocol for a Randomized Controlled Trial (REFUEL)
Source: JMIR Res Protoc. 2025 Aug 29;14:e69336. doi: 10.2196/69336 (PMC12432467; doi:10.2196/69336)
Supplement: Multimedia Appendix 5 [file resprot_v14i1e69336_app5.pdf]

STATISTICAL ANALYSIS PLAN (SAP) FOR THE REFUEL-TRIAL

Administrative information:

Sponsor name	Department of Oncology, Oslo University Hospital
Sponsor address	Oslo, Norway
REC no	153665
Trial title	Effects of a multidisciplinary intervention on fatigue and health-related quality of life aspects in lymphoma survivors with chronic fatigue (the REFUEL-trial). A randomized controlled trial
Trial ID	The REFUEL-trial
Trial registration number	NCT05130099

SAP and protocol version:

SAP version and date:	This SAP is version 1.0, dated February 25, 2025
Protocol version	This document has been written based on information contained in REC protocol version dated November 5, 2024 and the protocol article submitted December 3, 2024

SAP revision history:

Protocol version	SAP version	Section number changed	Description and reason for change	Date changed
[1.0]				

Følsomhet Intern (gul)

SIGNATURE PAGE

PRINCIPAL/COORDINATING INVESTIGATOR:

Lene Thorsen, Ph.D.

Department of Oncology and Department of Clinical Service, Oslo University Hospital, Oslo, Norway

Lene Thorsen

Signature

26.02.2025

Date (dd/mmm/yyyy)

TRIAL STATISTICIAN:

Torbjørn Wisløff, Ph.D
Health Services Research Unit, Akershus University Hospital, Lørenskog, Norway

Torbjørn Wisløff

Signature

25.02.2025

Date (dd/mmm/yyyy)

QC STATISTICIAN

Corina S. Rueegg, Ph.D.
Oslo Centre for Biostatistics and Epidemiology & Clinical Trials Unit, Oslo University Hospital, Oslo, Norway

Signature

25.02.2025

Date (dd/mmm/yyyy)

Følsomhet Intern (gul)

ABBREVIATIONS

ABVD	Adriamycin, Bleomycin Sulfate, Vinblastine Sulfate, Dacarbazine
AE	Adverse Event
BEACOPP	Bleomycin Sulfate
BMI	Body Mass Index
BFI-20	Big Five Inventory
CBT	Cognitive Behavioral Therapy
CF	Chronic Fatigue
CHOEP	Cyclophosphamide, Doxorubicin, Etoposide, Vincristine and Prednisone
CI	Confidence Interval
COPDAC	Cyclophosphamide, Oncovin, Prednisone, Dacarbazine
DCSQ	The Demand Control Support Questionnaire
DHAP	Dexamethasone, Cytarabine, Cisplatin
DL_{CO}	Diffusion capacity for carbon monoxide in the lung
EPOCH	Etoposide Phosphate, Prednisone, Oncovin, Cyclophosphamide, Hydroxydaunorubicin
EORTC QLQ-C30	European Organization for Research and Treatment of Cancer, Quality of Life Questionnaire-Core 30
FEV_1	Forced expiratory volume in the first second
FFQ	Food frequency questionnaire
FQ	Chalder Fatigue Questionnaire
FVC	Forced vital capacity
GAD-7	General Anxiety Disorder 7-items questionnaire
GMALL	The German Multicenter Acute Lymphoblastic Leukemia Protocol for Treatment of Adult Acute Lymphoblastic Leukemia
Gy	Gray
HDARAC	High-Dose Cytosine Arabinoside

Følsomhet Intern (gul)

HDMTX	High-Dose Methotrexate
HDT-ASCT	High-dose Therapy followed by Autologous Stem Cell Transplant
HRQoL	Health-related Quality of Life
HUNT	Trøndelag Health Study
IGEV	Ifosfamide, Gemcitabine, Vinorelbine
MVV	Maximal voluntary ventilation
OEPA	Oncovin, Etoposide, Prednisone, Adriamycin
OUS	Oslo University Hospital
PCS	Perceived Competence Scale
PHQ-9	Patient Health Questionnaire-9
R	Rituximab
R-IME	Rituximab, Ifosfamide, Methotrexate, Etoposide
RCT	Randomized Controlled Trial
SD	Standard Deviation
SpO_2	Oxygen saturation
SVS	Subjective Vitality Scale
SWLS	Satisfaction With Life Scale
$VEVCO_2$	Ventilatory efficiency slope
VO_2peak	Peak oxygen uptake
WAI	Work Ability Index

Følsomhet Intern (gul)

TABLE OF CONTENTS

1 INTRODUCTION ... 6
- 1.1 Background and Rationale ... 6
- 1.2 Intervention ... 7
- 1.3 Trial Objectives ... 7

2 TRIAL METHODS ... 8
- 2.1 Trial Design ... 8
- 2.2 Randomization and Blinding ... 8
- 2.3 Sample Size ... 9
- 2.4 Statistical Framework ... 9
- 2.5 Timing of Outcome Assessments ... 10
- 2.6 Statistical Interim Analyses and Stopping Guidance ... 10
- 2.7 Timing of Main Analysis ... 10

3 TRIAL POPULATION ... 11
- 3.1 Screening Data, Eligibility and Recruitment ... 11
- 3.2 Baseline Participant Characteristics ... 11
- 3.3 Withdrawal/Follow-up ... 13
- 3.4 Adherence and Protocol Deviations ... 13
- 3.5 Analysis Populations ... 14

4 OUTCOME DEFINITIONS ... 15
- 4.1 Primary Outcome ... 15
- 4.2 Secondary Outcomes ... 15
- 4.3 Primary Outcome Definition ... 18
- 4.4 Secondary Outcomes Definitions ... 18
- 4.5 Overview of Outcomes ... 19

5 ANALYSIS METHODS ... 23
- 5.1 Primary Outcome ... 23
- 5.2 Methods for Continuous Secondary Outcomes ... 24
- 5.3 Methods for Categorical Secondary Outcomes ... 28

6 SAFETY ANALYSES ... 28
- 6.1 Adverse Events ... 28

7 STATISTICAL SOFTWARE ... 28

8 REFERENCES ... 29

Følsomhet Intern (gul)

1 Introduction

This Statistical Analysis Plan (SAP) follows the "Guidelines for the Content of Statistical Analysis Plans in Clinical Trials" published by Gamble et al (December 19, 2017) in JAMA, complying with the ICH E9 guideline.

This SAP describes the details of the statistical analysis to estimate the efficacy of the randomized intervention on the primary and secondary endpoints of the REFUEL-trial. Efficacy of the outcomes will be distributed on five papers;

1) total fatigue (primary endpoint), mental and physical fatigue, health-related quality of life (HRQoL), vitality, life satisfaction, physical fitness and diet
2) mental health (symptoms of anxiety and depression)
3) work status and work ability
4) adherence to the Norwegian food-based dietary guidelines (intake of main food groups)
5) cardiorespiratory fitness /pulmonary function and exercise competence.

Not part of this SAP are the two sub-studies of the REFUEL-trial: 1) HRQoL among partners, and 2) outcomes among participants from follow-up questionnaires one and two years after the intervention period, as well as description of the statistical analyses of the cost-utility of the intervention.

1.1 Background and Rationale

Chronic fatigue (CF) is a subjective experience of substantial lack of energy, exhaustion and cognitive difficulties lasting for six months or longer [1, 2]. Between 15-40 % of lymphoma survivors are affected by CF, often for years beyond treatment completion [3-6]. It is one of the most distressing late effects after cancer, and limiting several aspects of daily life, including social participation, family and work life [7, 8]. In addition, CF may also place a considerable economic burden on the society due to reduction or loss of employment, as well as increased overall reliance on partners or caregivers [9, 10]. Despite the high prevalence and the huge negative consequences of CF, effective treatment and standardized follow-up care of CF are currently lacking.

The 5-year relative survival for Hodgkin lymphoma approaches 90%, and is about 70% for all non-Hodgkin lymphomas combined [11]. Since lymphoma survivors often receive their diagnosis at a young age, have a high cure rate, and a long life expectancy after extensive treatment, they are at high risk of CF [5, 11-16].

Currently, there are no effective medical treatments available for CF [17]. However, CF is associated with multiple modifiable behavioral factors, including emotional distress, physical inactivity, and unhealthy diets [18-22]. Exercise training and/or psychological interventions are therefore suggested as beneficial treatment options [23]. Several recent meta-analyses show that such interventions reduce fatigue during and after cancer treatment with small to moderate effect sizes [17, 24-26].

Low effect sizes may in part be attributed to not including participants screened for fatigue at inclusion and not defining fatigue as the primary outcome, and thus not testing interventions specifically tailored to reduce CF [27, 28]. Intervention studies with fatigue as the primary outcome including cancer survivors adequately screened for fatigue are therefore needed [17, 23, 26, 29].

Følsomhet Intern (gul)

Given the complex mechanisms underlying CF, it is plausible that the combination of several modalities (e.g. education, physical exercise, psychological strategies and nutrition) could have synergistic effects, leading to larger fatigue reductions compared to effects of single-focused interventions. This is the first RCT to examine the effect of a multidisciplinary intervention including patient education, physical exercise, a cognitive behavioral therapy (CBT) - based group program, and nutritional counselling on fatigue levels among lymphoma survivors with CF.

1.2 Intervention

1.2.1 Brief description of the study intervention

A 12-week multidisciplinary intervention including a combination of one digital session of patient education, two weekly physical exercise sessions (one supervised by local physiotherapist and one unsupervised), six weekly digital sessions of a CBT-based group program and three digital sessions of individual nutritional counselling.

1.2.2 Control settings

Usual care with no intervention. The usual care group was encouraged not to increase their physical activity level. After completion of the nine-month post-randomization assessments, participants randomized to the usual care group were offered a modified version of the intervention, according to their personally expressed needs.

1.3 Trial Objectives

1.3.1 Primary Objective

The primary objective of the REFUEL-trial is to examine the effect of a 12-week multidisciplinary intervention on the level of total fatigue at three-month post-randomization, compared to usual care, among lymphoma survivors with CF.

1.3.2 Secondary Objectives

The secondary objectives are:

- To examine the effect of the intervention on:
    - Level of total fatigue at six- and nine-month post-randomization
    - Level of physical and mental fatigue, HRQoL, vitality, life satisfaction, symptoms of depression and anxiety, work status, work ability, diet and nutrition, body mass index, exercise competence and physical fitness at three-, six- and nine-month post-randomization

Følsomhet Intern (gul)

2 Trial Methods

2.1 Trial Design

The REFUEL-trial is a multicenter, 1:1 parallel-arm, superiority RCT including lymphoma survivors from Oslo University Hospital (OUS) and St. Olav's Hospital (Figure 1). Participants are randomized to the intervention group or usual care group after responding to questionnaires and undergo physical tests at baseline (T0). The intervention lasts for 12 weeks. Both groups undergo the same assessments at three- (T1), six- (T2), and nine-month (T3) post-randomization. At T3, the usual care group is offered a modified version of the intervention program. Participants also completed follow-up questionnaires at one and two years follow-up (15- and 27-month post-randomization). The results of this follow-up are not part of the current SAP.

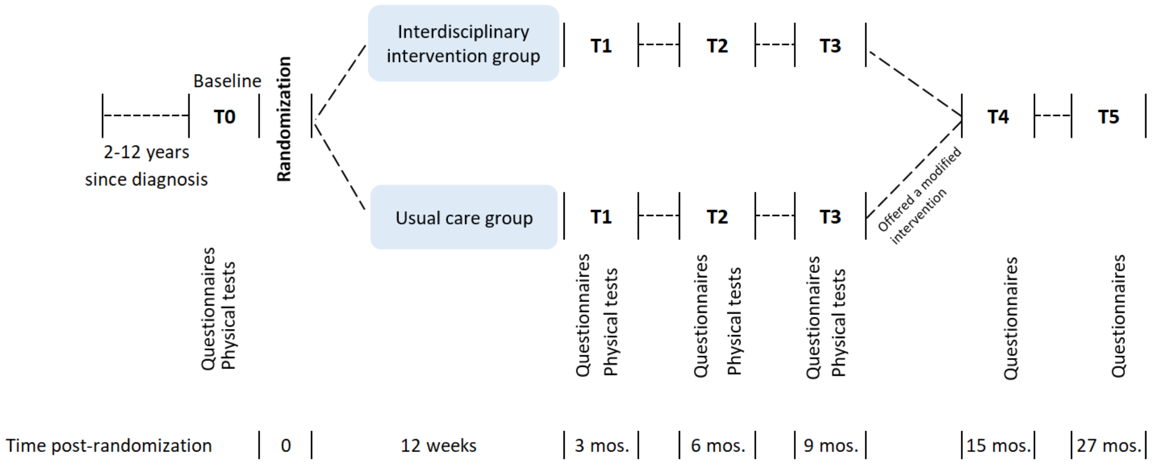

Figure 1: REFUEL study design. Displays the design and assessments time points/methods in the REFUEL-trial. T0: baseline tests prior to randomization (week 0), T1: assessments at three-month post-randomization. T2: assessments at six-month post-randomization. T3: assessments at nine-month post-randomization. T4: assessments at 15-month post-randomization. T5: assessments at 27- month post-randomization.

2.2 Randomization and Blinding

Participants were allocated in a 1:1 ratio between the intervention group and usual care group using a block randomization procedure with random block sizes of n=2 and n=4. A computer-generated randomization list was generated using Stata statistical software (version 17.0; Stata Corporation). The allocation sequence was prepared by an independent statistician.

Details of block size and allocation sequence generation were unavailable to those who enrolled patients or assign study group.

Due to the nature of the intervention, participants, physiotherapists/study personnel delivering the intervention and assessors were not blinded to group assignment after randomization. The study statistician was blinded to group allocation when writing the SAP and programming the analyses.

Følsomhet Intern (gul)

2.3 Sample Size

Sample size calculation was based on the primary outcome, i.e. change in total fatigue measured by Chalder Fatigue Questionnaire (FQ) from pre- to post-intervention. Based on a pilot study by Oldervoll et al [30] on an exercise intervention among lymphoma survivors with CF and our feasibility study [31], we assumed that the baseline level of fatigue would be 21.0 in the total study sample.

The minimally important difference in total fatigue has not been defined for Chalder FQ [1] in cancer populations. However, in previous research among cancer survivors, a minimally important difference in HRQoL outcomes has been defined as a 10 % difference on a 0-100 scale [32]. Likewise the REFUEL-trial was powered to detect a 10 % difference between groups at T1, i.e. mean difference of 3.3 points (standard deviation (SD) 6.1) in total fatigue score (on a 0-33 scale). We assumed no change in the usual care group (T1 level of fatigue 21.0, SD 6.1), and 3.3 points reduction in the intervention group (T1 level of fatigue 17.7, SD 6.1). A 3.3 point difference also corresponds to reported minimally important differences in FQ in other patient populations [33].

With a two-sided significance level of 5 % and a power of 80 %, 54 survivors were required in each group. To have a sufficient sample size to also detect a 10 % group difference in the secondary HRQoL outcomes with similar assumptions, 62 survivors were needed in each group. Assuming a 20 % loss to follow-up, we aimed to recruit 74 survivors in each group.

2.4 Statistical Framework

2.4.1 Hypothesis Test

This REFUEL-trial is designed to establish the superiority of a multidisciplinary intervention compared to usual care on change in total fatigue at three-month post-randomization.

The primary null hypothesis is that there is no difference in the total fatigue score at three-month post-randomization between the two arms.

The primary alternative hypothesis is that there is a difference in the total fatigue score at three-month post-randomization between the two arms.

This trial tests one primary hypothesis. All other efficacy analyses will be regarded as supportive or exploratory.

2.4.2 Confidence Intervals and p-values

All calculated p-values will be two-sided and compared to a 5% significance level. If a p-value is less than 0.05, the corresponding treatment group difference will be denoted as statistically significant. All efficacy estimates will be presented with two-sided 95% CIs. As there is only one primary null hypothesis to be tested in this trial, there will be no adjustments for multiplicity. For Paper 1, p-values will only be presented to indicate statistical significance for the primary outcome, while all other analyses will only be presented with 95 % CIs. For the next papers, p-values will be presented to indicate statistical significance for the main outcomes.

2.4.3 Decision Rule

This trial is designed to address a single primary outcome. Superiority is claimed if the primary null hypothesis is rejected on the significance level (alpha) of 0.05 (two-sided).

Følsomhet Intern (gul)

2.5 Timing of Outcome Assessments

For all questionnaires and physical tests, assessments should occur within a time window of the scheduled assessment time point. Assessments outside this time window is regarded a protocol deviation. The target day and visits window is defined in the protocol as:

**Visit Label**	**Target Day**	**Target week**	**Definition (Day window)**	**Lower limit (incl.)**	**Upper limit (incl.)**
Medical screening	< -1				
T0. Randomization	0	0	0	0	0
T1. Three-month post-randomization	91(98[a])	13(14[a])	Target day -7(+14 days[b])	84(91[a])	105(112[a])
T2. Six-month post-randomization	175(182[a])	25(26[a])	Target day -7(+28 days)	168(175[a])	203(210[a])
T3. Nine-month post-randomization	259(266[a])	37(38[a])	Target day -7(+28 days)	252(259[a])	287(294[a])

[a]For participants who had the intervention interrupted by holidays (i.e. Christmas or Easter), the time from T0 to T1 was extended with 7 days

[b]DIGIKOST: + 28 days (because participants report their typical average diet the last 2 weeks in DIGIKOST)

In the per protocol analyses, assessments completed outside the scheduled time window will be excluded.

2.6 Statistical Interim Analyses and Stopping Guidance

There was no interim efficacy analysis performed for this trial.

2.7 Timing of Main Analysis

The main analysis will be performed when all participants have completed the nine-month post-randomization assessments. All data will be entered in the study database, verified and validated, the SAP finalized and signed, and the primary database will be locked. An exception is the results on diet, of which the three-month post-randomization linear regression analysis was performed before the SAP was signed and database locked.

Følsomhet Intern (gul)

3 Trial Population

3.1 Screening Data, Eligibility and Recruitment

The total number of screened survivors and reasons for not entering the trial or lost to follow-up will be summarized and tabulated.

A CONSORT flow diagram will be used to summarise the number of survivors who:

- were assessed for eligibility in the registries at OUS and St. Olav's Hospital
- were eligible from registries (invited)
- were ineligible from registries*
- declined the invitation, did not respond and the number of invitations that came in return
- responded and were ineligible*
- were eligible and randomized
- were eligible but not randomized*
- received the randomized allocation
- did not receive the randomized allocation*
- were lost to follow-up*
- discontinued the intervention*
- were randomized and included in the primary analysis
- were randomized and excluded from the primary analysis*

*reasons will be provided.

3.2 Baseline Participant Characteristics

The following demographics and baseline characteristics will be summarised:

- Age in years at inclusion[1]
- Sex[1]
- Living status[1]
- Education level[1]
- Work status[1]
- Age in years at diagnosis[2]
- Years since diagnosis[2]
- Lymphoma subtype: Hodgkin lymphoma, diffuse large b-cell lymphoma, primary mediastinal b-cell lymphoma, Burkitt lymphoma, T cell/histiocyte-rich large B-cell lymphoma or T cell lymphoblastic lymphoma[2]
- Stage at diagnosis: I, II, III or IV[2]
- Presence of B-symptoms at diagnosis[2]
- Primary chemotherapy regimen[2]: number of cycles and combinations of ABVD, BEACOPP, OEPA, COPDAC, CHO(E)P, EPOCH, HDMTX, HDARAC, GMALL, Hammersmith-regimen, HDT-ASCT
- Radiotherapy[2] (median dose in Gy, field)
- Surgery[2]
- Relapse[2]

Følsomhet Intern (gul)

- Chemotherapy regimen for progression or relapse[2]: HDT-ASCT, R-IME, DHAP, IGEV
- Comorbidities[2]
- Personality traits self-reported by BFI-20[3]
- Work environment self-reported by The Demand Control Support Questionnaire[4]
- Physical activity level self-reported by Godin Leisure-Time Exercise Questionnaire (GLTEQ)[5]
- Alcohol consumption, smoking, snuff[6]
- Sleep problems[7]

***Abbreviations**: ABVD: Adriamycin, Bleomycin Sulfate, Vinblastine Sulfate, Dacarbazine. BEACOPP: Bleomycin Sulfate, Etoposide Phosphate, Adriamycin, Cyclophosphamide, Oncovin, Procarbazine Hydrochloride, Prednisone. OEPA: Oncovin, Etoposide, Prednisone, Adriamycin. COPDAC: Cyclophosphamide, Oncovin, Prednisone, Dacarbazine. CHOEP: Cyclophosphamide, Doxorubicin, Etoposide, Vincristine and Prednisone. R: Rituximab. EPOCH: Etoposide Phosphate, Prednisone, Oncovin, Cyclophosphamide, Hydroxydaunorubicin. HDMTX: High-Dose Methotrexate. HDARAC: High-Dose Cytosine Arabinoside. GMALL: the German Multicenter Acute Lymphoblastic Leukemia (GMALL) Protocol for Treatment of Adult Acute Lymphoblastic Leukemia. HDT-ASCT: High-dose Therapy (HDT) followed by Autologous Stem Cell Transplant (ASCT). Gy: Gray. R-IME: Rituximab, Ifosfamide, Methotrexate, Etoposide. DHAP: Dexamethasone, Cytarabine, Cisplatin. IGEV: Ifosfamide, Gemcitabine, Vinorelbine*

[1]Socio-demographic variables are self-reported by questions used in the HUNT-study [34].

[2]Cancer-related variables and comorbidities are extracted from the medical record and the medical screening.

[3]Personality is self-reported using a short version of the Big Five Inventory (BFI-20) [35]. The BFI-20 measures the five personality domains openness, conscientiousness, extraversion, agreeableness, and neuroticism. The questionnaire consists of 20 items, distributed on four items for each personality domain. Items are scored on a Likert scale from 1 (the item does not describe the respondent at all) to 7 (the item describes the respondent very well) and aggregated into a sum score ranging from 4 to 28 for each personality domain.

[4]Work environment is self-reported by the The Demand Control Support Questionnaire (DCSQ) [36]. The DCSQ includes 17 statements concerning psychological demands (five items), decision latitude (six items), and social support (six items) at the workplace. The level of agreement with these statements is reported on a four-point scale ranging from complete agreement (1) to no agreement at all (4), which are aggregated into sum scores ranging from 5 to 20 for psychological demands and 6 to 24 for decision latitude and social support at the workplace. Higher scores indicate higher psychological demands, decision latitude and social support at work.

[5]Physical activity level is self-reported using a modified version of the Godin Leisure-Time Exercise Questionnaire (GLTEQ) [37]. The GLTEQ assesses the average frequency and duration of mild, moderate and vigorous leisure-time physical activity during a typical week. By summing the weekly minutes of moderate and/or vigorous intensity levels of physical activity participants can be dichotomized into meeting or not meeting the World Health Organization guidelines on physical activity (i.e. 150 to 300 minutes of moderate intensity or 75 to 150 minutes of vigorous intensity per week, or an equivalent combination of moderate- and vigorous-intensity activity throughout the week) [38]. The metabolic equivalent (MET)-hours and proportion of participants meeting strength exercise guidelines [38] will also be reported.

[6]Smoking (never/former/sometimes/daily), snuff (never/former/sometimes/daily), and frequency of alcohol consumption (not the last year/ once or less per month/2-4 times per month/4 times or more per month/2-3 times per week/never) are measured by the same questions as used in HUNT 4-study [34] .

[7]Sleep problems are assessed by the following question extracted from the HUNT 4-Study [34]: How often in the last three months have you experienced: difficulty falling asleep/waking up repeatedly through the night/ woken too early and could not get back to sleep/difficulty coping during the daytime (socially or professionally)

Følsomhet Intern (gul)

due to sleep problems. Participants are classified as having sleep problems if they answer 'several times a week' on at least one night-time symptom and difficulty coping during the day due to sleep problems.

Baseline characteristics will be summarised by randomized group using descriptive statistics (N, mean, standard deviation, median, 25/75 percentiles, minimum, and maximum for continuous variables, and number and percentages for categorical variables). There will be no statistical analysis of difference between groups in baseline characteristics. Any clinical important imbalance between the intervention and usual care groups will be noted.

3.3 Withdrawal/Follow-up

The status of eligible and randomized patients at trial end will be tabulated by group according to

- completed intervention and assessments
- completed assessments, but not intervention
- withdrew consent
- lost to follow-up

3.4 Adherence and Protocol Deviations

3.4.1 Adherence to the Multidisciplinary Intervention

Adherence to the patient education, exercise sessions, CBT-based group sessions and nutrition counselling sessions is assessed by calculating the attendance rate for each of the components, defined as:

% adherence = (number of sessions attended / number of sessions offered) * 100

For each part of the intervention, a participant is considered adherent if attending at least 65 % of the offered sessions (i.e. 16 of 23 exercise sessions (70 %), four of six CBT-based group sessions (67 %) and two of three nutritional counselling sessions (67 %)). For the total intervention, a participant is defined as adherent if they have attended the patient education and at least 65 % of each of the other parts of the intervention (i.e. the combination of attending the patient education, ≥ 16 exercise sessions, ≥ four CBT-sessions and ≥ two nutritional counseling sessions).

Adherence to the exercise program is assessed based on information in the exercise log registrations. In addition, we will provide detailed information about the exercise frequency and duration, and the total number of aerobic sessions completed at the planned intensity, below or above the planned intensity (% of HRpeak), but this is not taken into consideration when calculating adherence.

A participant is considered adherent to usual care if their VO_2peak or self-reported physical activity level increased by less than two times the standard deviation of the mean change in the intervention group.

3.4.2 Protocol Deviations

The following are protocol deviations regarded to affect the efficacy of the intervention:

- Entering the trial when the eligibility criteria should have prevented trial entry
- Not being adherent to the allocated intervention as defined above (point 3.4.1)
- Completing tests outside visit window described in point 2.5

Følsomhet Intern (gul)

The number (and percentage) of patients with any deviations will be summarised by intervention/usual care group with details of type of deviation provided. The number and percentage of adherent and non-adherent participants will be summarised for each part of the intervention and the total intervention. The patients that are included in the Full Analysis Set (FAS; see below) will be used as the denominator to calculate the percentages. No formal statistical testing will be undertaken.

3.5 Analysis Populations

The Enrolled set will include all patients who have provided informed consent and have been included into the study data base.

The Full Analysis Set (FAS) will be defined as all participants randomly assigned to one of the two groups that were not wrongly included in the study and had valid baseline measure of the primary outcome.

The Safety Analysis Set will include all patients having participated in at least one of the intervention sessions (patient education, exercise, cognitive behavioural therapy-based group program or individual nutritional counselling) for the participants in the intervention group and having been told to maintain their usual physical activity level for participants of the usual care group.

The Per Protocol Analysis Set 1 (PPS1) will include all randomized patients with none of the protocol deviations defined above (point 3.4.2).

Other per protocol analysis sets are defined as follows:

- PPS2x: Same as PPS1, but the requirement of adherence to at least 65% of the intervention is only required for adherence to the CBT-program and exercise program
- PPS3x: Same as PPS1, but the requirement of adherence to at least 65% of the intervention is only required for adherence to the exercise program
- PPS4x: Same as PPS1, but the requirement of adherence to at least 65% of the intervention is only required for adherence to the nutrition counselling

Følsomhet Intern (gul)

4 Outcome Definitions

4.1 Primary Outcome

4.1.1 Level of Total Fatigue

Level of total fatigue is self-reported by the Chalder FQ [1]. The questionnaire consists of 11 items distributed on a physical (seven items) and a mental (four items) fatigue scale. Each item has four response categories scored from 0 ("less or better than usual/not at all" to 3 ("a lot more than usual"). The total fatigue score is the sum of all 11 items (0 to 33). Higher scores imply more fatigue.

In the case of missing items, scale scores are set to missing, and handled by the mixed model for those with a valid baseline value.

4.2 Secondary Outcomes

4.2.1 Level of Mental Fatigue

Level of mental fatigue is self-reported by the Chalder FQ [1] (see above for scoring instructions). Level of mental fatigue is summed by the four items on the mental fatigue subscale, giving a total mental fatigue score between 0-12.

4.2.2 Level of Physical Fatigue

Level of physical fatigue is self-reported by the Chalder FQ [1] (see above for scoring instructions). Level of physical fatigue is summed by the seven items on the physical fatigue subscale, giving a total physical fatigue score between 0-21.

4.2.3 Health-Related Quality of Life

Health-related quality of life is self-reported by the EORTC QLQ-C30 questionnaire [39]. This questionnaire includes five functioning scales (physical, role, cognitive-, emotional- and social function), three symptom scales (fatigue, pain, and nausea/vomiting), a global health status/QoL scale and six single items (dyspnoea, insomnia, appetite loss, constipation, diarrhoea, financial difficulties).

Items in the functioning scales, symptom scales and single items are coded with response categories 1 (not at all), 2 (a little), 3 (quite a bit) and 4 (very much). The two items measuring global health status/QoL are rated on a Likert scale from 1 (very poor) to 7 (excellent).

All the scales are linearly transformed to a scale of 0 to 100 according to the EORTC scoring manual (Microsoft Word - SCmanual.doc) and, if applicable, aggregated as average into the specific scales. Increasing scores on functioning scales and global health status/QoL imply better functioning/QoL, while increasing scores on symptom scales and single items imply more severe symptoms [39].

Calculation of the 0 to 100 score is done as follows:

1. Estimate the raw score by averaging the items that contribute to the scale.
2. Use a linear transformation to standardise the raw score, so that scores range from 0 to 100; a higher score represents a higher ("better") level of functioning, or a higher ("worse") level of symptoms.

Følsomhet Intern (gul)

The procedure is shown in Box 1 (extracted from the EORT QLQ-C30 scoring manual):

For all scales, the *RawScore*, *RS*, is the mean of the component items:

$$RawScore = RS = (I_1 + I_2 + ... + I_n)/n$$

Then for **Functional scales**:

$$Score = \left\{1 - \frac{(RS-1)}{range}\right\} \times 100$$

and for **Symptom scales / items** and **Global health status / QoL:**

$$Score = \{(RS-1)/range\} \times 100$$

Box 1: Calculation of scales scores in EORTC QLQ-C30

The range is the difference between the maximum and the minimum possible value of the raw score. Most items are scored 1 to 4, giving range = 3. The exception is the items contributing to the global health status/QoL, which are 7-point questions with a range of 6.

Missing values will be handled according to the EORTC QLQ-C30 scoring manual: if at least half of the items from the scale has been answered, all the items that were completed are used, and the standard equations for calculating the scale scores are applied (items with missing values are ignored when making the calculations). For single items, scores are set to missing.

In addition to EORTC QLQ-C30, the participants' health status and HRQoL is self-reported using the by EQ-5D-5L questionnaire [40]. The questionnaire consists of the five dimensions mobility, self-care, usual activities, pain and discomfort, anxiety and depression. Each dimension has five severity levels that are described by statements appropriate to that dimension, coded from 1 (no problems) to 5 (unable to perform activities/extreme level of symptoms). Answers to the five questions are combined into an overall measure of HRQoL with values up to a maximum of 1 using a recent Norwegian valuation study [41]. This measure will inform the economic evaluation of cost per quality-adjusted life years. The participants also rate their health status from 0 (the worst health you can imagine) to 100 (the best health you can imagine).

In the case of missing items, scale scores are set to missing, and handled by the mixed model for those with a valid baseline value.

4.2.4 Life Satisfaction

Life satisfaction is self-reported using the Satisfaction With Life Scale (SWLS) [42]. The scale consists of five items with statements regarding life satisfaction, scored from 1 (totally disagree) to 7 (totally agree). A scale score is formed by averaging the individuals' items scores. Increasing scores imply higher satisfaction with life. In the case of missing items, scale scores are set to missing, and handled by the mixed model for those with a valid baseline value.

4.2.5 Subjective Vitality

Subjective vitality is self-reported by the five item version of the Subjective Vitality Scale (SVS) [43]. The scale consists of five items about level of energy and vitality scored from 1 (totally disagree) to 7 (totally agree). A scale score is formed by averaging the individuals' items scores. Increasing scores reflect higher subjective vitality. In the case of missing items, scale scores are set to missing, and handled by the mixed model for those with a valid baseline value.

Følsomhet Intern (gul)

4.2.6 Symptoms of Anxiety

Symptoms of anxiety are self-reported by the General Anxiety Disorder 7-items questionnaire (GAD7) [44]. The questionnaire consists of seven items scored from 0 to 3, which are aggregated into a total score from 0 to 21. Higher scores imply higher level of anxiety symptoms. In the case of missing items, scale scores are set to missing, and handled by the mixed model for those with a valid baseline value.

4.2.7 Symptoms of Depression

Symptoms of depression are self-reported using the Patient Health Questionnaire-9 (PHQ-9) [45]. The PHQ-9 questionnaire consists of nine items scored from 0 to 3, aggregated into a total score from 0 to 27. A higher score implies a higher level of depressive symptoms. In the case of missing items, scale scores are set to missing, and handled by the mixed model for those with a valid baseline value.

4.2.8 Work Status

Work status is self-reported by questions on current work situation, from the Trøndelag Health Study (HUNT 4) [34]. Response options include: working full-time, working part-time, job-seeking, laid off, sick leave, work assessment allowance, disability pension, self-employed, retired, military service/education, homemaker, and other. For analyses, this will be transformed to a binary variable indicating work status.

4.2.9 Work Ability

Work ability is self-reported using three items from the original seven-items Work Ability Index (WAI) [46]. The WAI includes both physical and psychological work ability in relation to demands of the job rated by the following response alternatives: very good/rather good/moderate/rather poor/very poor. Current work ability is assessed by the Work Ability Score (WAS), which includes current overall work ability compared to the participant’s lifetime best work ability on a scale from 0 (extinguished work ability) to 10 (highest possible work ability). In the case of missing items, scale scores are set to missing, and handled by the mixed model for those with a valid baseline value.

4.2.10 Diet

Dietary intake is self-reported using the digital food-frequency questionnaire DIGIKOST-FFQ [47-50]. The DIGIKOST-FFQ is a digital diet and lifestyle assessment tool designed to measure adherence to the Norwegian food-based dietary guidelines [51]. To measure efficacy on diet the following outcomes from DIGIKOST-FFQ will be used:

- Intake (in grams per day) of the main food groups, including fruits and berries, vegetables, whole grain products, fish (fatty and lean fish), unsalted nuts, dairy products (high- and low-fat content), meat (red and processed meat), foods rich in sugars and fat and sugar-rich beverages.
- The Norwegian diet index, which consists of 12 components corresponding to the Norwegian food–based dietary guidelines with a 3-level scoring approach including three categories representing low, intermediate, and high adherence. Each of the components is assigned a value of 0, 0.5 or 1 corresponding to low, intermediate and high adherence, except for plant-based foods, which is assigned a value of 0, 1.5 or 3, giving a composite diet index ranging in scores from 0 (lowest adherence) to 20 (highest adherence) points[50].
- The Norwegian lifestyle index consists of the five components diet, physical activity, normal weight, alcoholic drinks, and tobacco use. Each component is assigned a value of 0, 0.5 or 1, giving a final score ranging from zero to five points [50].

Følsomhet Intern (gul)

4.2.11 Body Mass Index

Body Mass Index (BMI) [kg/m^2] is calculated as body weight in kilograms divided by the square of the height in meters. Weight and height was measured at the physical tests, and was also self-reported in DIGIKOST-FFQ.

4.2.12 Exercise Competence

Exercise competence is self-reported using an exercise version of the 4-item Perceived Competence Scale (PCS) [52]. The participants respond to four statements related to their ability to be physically active on a 7-point Likert scale ranging from 1 (not at all true) to 7 (very true). The 4 items are averaged into a sum score ranging from 1 to 7. A higher averaged sum of the four items reflects higher perceived exercise competence. In the case of missing items, scale scores are set to missing, and handled by the mixed model for those with a valid baseline value.

4.2.13 Cardiorespiratory Fitness/pulmonary function

Cardiorespiratory fitness will be expressed by peak oxygen uptake (VO_2peak, $mL \cdot kg^{-1} \cdot min^{-1}$)), assessed by CPET. For participants who were unable to undergo CPET, a VO_2peak prediction equation [53] is applied based on the results (i.e. maximum speed, grade, as well as age and body weight) of an indirect treadmill test using the same modified Balke protocol as for CPET.

Pulmonary function is expressed as forced vital capacity (FVC), forced expiratory volume in the first second (FEV_1), maximal voluntary ventilation (MVV) and diffusion capacity for carbon monoxide in the lung (DL_{CO}).

4.2.14 Muscle Strength

Muscle strength is assessed by a one-repetition maximum (1RM) test, i.e. the maximal workload in kg that can be lifted once, in leg press. For the upper body, muscle strength is assessed by the maximum number of push-ups performed in one set.

4.3 Primary Outcome Definition

The primary outcome is the difference in total level of fatigue at three-month post-randomization (T1). The primary outcome is continuous.

4.4 Secondary Outcomes Definitions

The continuous secondary outcomes (see table below) are defined as the difference in the respective outcomes at three-month post-randomization (T1), and six- (T2) and nine-month post-randomization (T3). Work status is the only categorical variable, defined as the difference in proportion of participants working or not at three-month post-randomization (T1), and six- (T2) and nine-month post-randomization (T3).

Følsomhet Intern (gul)

4.5 Overview of Outcomes

Level	Outcome	Paper	Timeframe	Type
Primary	Level of total fatigue, FQ	1	T0-T1	Continuous
Secondary	Level of total fatigue, FQ	1	T0-T2-T3	Continuous
Secondary	Level of physical fatigue, FQ	1	T0-T1-T2-T3	Continuous
Secondary	Level of mental fatigue, FQ	1	T0-T1 - T2-T3	Continuous
Secondary	Physical functioning, EORTC QLQ-C30	1	T0-T1-T2-T3	Continuous
Secondary	Role functioning, EORTC QLQ-C30	1	T0-T1-T2-T3	Continuous
Secondary	Cognitive functioning, EORTC QLQ-C30	1	T0-T1-T2-T3	Continuous
Secondary	Emotional functioning, EORTC QLQ-C30	1	T0-T1-T2-T3	Continuous
Secondary	Social functioning, EORTC QLQ-C30	1	T0-T1-T2-T3	Continuous
Secondary	Fatigue, EORTC QLQ-C30	1	T0-T1-T2-T3	Continuous
Secondary	Nausea and vomiting, EORTC QLQ-C30	1	T0-T1-T2-T3	Continuous
Secondary	Pain, EORTC QLQ-C30	1	T0-T1-T2-T3	Continuous
Secondary	Dyspnoea, EORTC QLQ-C30	1	T0-T1-T2-T3	Continuous
Secondary	Insomnia, EORTC QLQ-C30	1	T0-T1-T2-T3	Continuous
Secondary	Appetite loss, EORTC QLQ-C30	1	T0-T1-T2-T3	Continuous

Secondary	Constipation, EORTC QLQ-C30	1	T0-T1-T2-T3	Continuous
Secondary	Diarrhoea, EORTC QLQ-C30	1	T0-T1-T2-T3	Continuous
Secondary	Financial difficulties, EORTC QLQ-C30	1	T0-T1-T2-T3	Continuous
Secondary	Global health status/QoL, EORTC QLQ-C30	1	T0-T1-T2-T3	Continuous
Secondary	Health status from 0 to 100, EQ-5D-5L	1	T0-T1-T2-T3	Continuous
Secondary	Life Satisfaction, SWLS	1	T0-T1-T2-T3	Continuous
Secondary	Vitality, SVS	1	T0-T1-T2-T3	Continuous
Secondary	Estimated VO_{2peak} ($mL \cdot kg^{-1} \cdot min^{-1}$)	1	T0-T1-T2-T3	Continuous
Secondary	Muscle strength legs in leg press (kg)	1	T0-T1-T2-T3	Continuous
Secondary	Muscle strength upper body (number of push-ups repetitions)	1	T0-T1-T2-T3	Continuous
Secondary	Diet Index, DIGIKOST-FFQ	1	T0-T1-T2-T3	Continuous
Secondary	Symptoms of anxiety, GAD-7	2	T0-T1-T2-T3	Continuous
Secondary	Symptoms of depression, PHQ-9	2	T0-T1-T2-T3	Continuous
Secondary	Work status	3	T0-T1-T2-T3	Categorical
Secondary	Work ability, WAI	3	T0-T1-T2-T3	Continuous
Secondary	Mobility, EQ-5D-5L	3	T0-T1-T2-T3	Continuous
Secondary	Self-care, EQ-5D-5L	3	T0-T1-T2-T3	Continuous
Secondary	Usual activities, EQ-5D-5L	3	T0-T1-T2-T3	Continuous

Følsomhet Intern (gul)

Secondary	Pain and discomfort, EQ-5D-5L	3	T0-T1-T2-T3	Continuous
Secondary	Anxiety and depression, EQ-5D-5L	3	T0-T1-T2-T3	Continuous
Secondary	Overall measure of HRQoL with values up to a maximum of 1, EQ-5D-5L	3	T0-T1-T2-T3	Continuous
Secondary	Overall measure on health status/HRQoL, EQ-5D-5L	3	T0-T1-T2-T3	Continuous
Secondary	Intake of fruits and berries (grams)	4	T0-T1-T2-T3	Continuous
Secondary	Intake of unsalted nuts (grams)	4	T0-T1-T2-T3	Continuous
Secondary	Intake of vegetables (grams)	4	T0-T1-T2-T3	Continuous
Secondary	Intake of whole grain products (grams)	4	T0-T1-T2-T3	Continuous
Secondary	Intake of fish (total and fatty fish) (grams)	4	T0-T1-T2-T3	Continuous
Secondary	Intake of dairy products (low-fat and high-fat) (grams)	4	T0-T1-T2-T3	Continuous
Secondary	Intake of meat (red- and processed meat) (grams)	4	T0-T1-T2-T3	Continuous
Secondary	Intake of sugar-rich beverages (grams)	4	T0-T1-T2-T3	Continuous
Secondary	Intake of sugar- and fat-rich foods (grams)	4	T0-T1-T2-T3	Continuous

Følsomhet Intern (gul)

Secondary	Lifestyle index, DIGIKOST-FFQ	4	T0-T1-T2-T3	Continuous
Secondary	Body mass index (kg/m^2)	4	T0-T1-T2-T3	Continuous
Secondary	FEV_1	5	T0-T1	Continuous
Secondary	FVC	5	T0-T1	Continuous
Secondary	MVV	5	T0-T1	Continuous
Secondary	DL_{CO}	5	T0-T1	Continuous
Secondary	Directly assessed VO_2peak, ($mL \cdot kg^{-1} \cdot min^{-1}$)	5	T0-T1-T2-T3	Continuous
Secondary	HRpeak ($s \cdot min^{-1}$)	5	T0-T1	Continuous
Secondary	SpO_2	5	T0-T1-T2-T3	Continuous
Secondary	Breathing reserve, %	5	T0-T1-T2-T3	Continuous
Secondary	$VEVCO_2$ slope	5	T0-T1-T2-T3	Continuous
Secondary	Exercise Competence, PCS	5	T0-T1-T2-T3	Continuous

5 Analysis Methods

After the SAP is signed, the statistician gains access to the data file without group affiliation to check if the assumptions for the linear mixed model analyses are met. Thereafter, the statistician gains access to the main file with group affiliation.

5.1 Primary Outcome

5.1.1 Primary Analysis

The primary analysis will be performed in the FAS (intention-to-treat (ITT) approach). The primary outcome will be analyzed with a linear mixed model for repeated measures with level of total fatigue score at T1, T2 and T3 as dependent variable, with random effects for hospital (OUS and St. Olavs) and person, a treatment (intervention and usual care group) * timepoint interaction, and fixed effects for baseline FQ-score. Time will be entered as categorical variable (T1, T2 and T3).

The primary effect estimate will be the difference in the adjusted marginal means for total FQ-score comparing the intervention and usual care group at T1, extracted from the mixed model using the delta method. The marginal mean difference is based on a counterfactual prediction of the mean in each person under each treatment option. The primary estimate will be accompanied by a two-sided 95% confidence interval and p-value.

Missing total fatigue score at T1, T2 or T3 will be handled by the mixed model using a corrected maximum likelihood approach.

5.1.2 Secondary Analysis

Same analysis as the primary, but in the per protocol set 1 (point 3.5).

5.1.3 Sensitivity analyses

We will perform the following sensitivity analyses in the FAS:

- Same as primary analysis, but without hospital in the mixed model
- Same as primary analysis, but using multiple imputation with chained equations to impute missing total fatigue score at T1, T2 or T3, if > 10 % in at least one of the groups have missing scale scores (given that < 50 % of the items are missing).
- Analyzing the change from T0 to T1 in the total fatigue score as linear regression model with total fatigue score at T1 as dependent variable and including treatment, hospital and total fatigue score at T0 as covariates.

5.1.4 Assumption Checks and Alternative Analyses

We will do the following checks for the linear mixed model:

- Check for linearity of the residuals by a qq-plot of the residuals

If the plot indicate severe deviations from assumptions for linear mixed model, we will perform alternative analyses testing in the following order: gaussian family with log link, gamma family with identity link, gamma family with log link, gaussian family with inverse link, gamma family with inverse link.

5.1.5 Subgroup Analyses

We will do the following subgroup analyses in the FAS to investigate whether the treatment effect differs for certain subgroups. The analysis will be performed by adding the respective variable to the treatment*time interaction in the mixed model described as primary analysis:

- Sex (male, female)

Følsomhet Intern (gul)

- Age at inclusion (<median, ≥median)
- Years from diagnosis (<median, ≥median)
- Living with children (yes, no)
- Fulltime work (yes, no)
- Education (high, low)
- Number of chemotherapy cycles (2-4, >4 cycles)
- Lymphoma subtype (Hodgkin, non-Hodgkin)
- Radiation therapy (yes, no)
- Baseline level of total fatigue (divided into tertiles)
- Adherence to the total intervention (completing ≥ 70 % of the exercise program and 65% of the nutrition counselling and CBT-sessions versus not)
- Adherence to the CBT-sessions (completing ≥65 % of the CBT-sessions versus not)
- Adherence to the exercise-program (completing ≥70 % of the exercise program versus not)

The stratified treatment effect (marginal mean difference at T1 with 95% CIs and p-value of the interaction) by subgroup will be presented, e.g. by using a forest plot.

5.2 Methods for Continuous Secondary Outcomes

5.2.1 Primary Analysis (ITT)

See table under section 4.4 for an overview of the secondary outcomes referred to. Continuous secondary outcomes will be analyzed in the same way as the primary outcome (point 5.1).

5.2.2 Secondary Analysis (per protocol)

Same analysis as the primary, but using per protocol analysis sets as defined in the table below.

Per protocol set	Adherence to	Outcomes
PPS1	• Patient education<br>• Exercise program<br>• CBT program<br>• Nutrition counselling	• mental fatigue<br>• physical fatigue<br>• EORTC QLQ-C30 outcomes<br>• satisfaction with life<br>• subjective vitality<br>• work status<br>• work ability<br>• EQ-5D-5L outcomes
PPS2x	• CBT program<br>• Exercise program	• anxiety symptoms<br>• depressive symptoms
PPS3x	• Exercise program	• VO_2peak<br>• muscle strength
PPS4x	• Nutrition counselling	• diet index<br>• lifestyle index<br>• daily intake of main food groups

Følsomhet Intern (gul)

For all per protocol analyses the usual care participants are excluded if they increase their VO_2peak or self-reported physical activity level by more than two times the standard deviation of the mean change in the intervention group.

5.2.3 Sensitivity Analysis

For selected secondary outcomes, we will use multiple imputation with chained equations to impute missing scores at T1, T2 or T3, if > 10 % in at least one of the groups have missing scale scores (given that < 50 % of the items are missing).

5.2.4 Assumption Checks and Alternative Analyses

For linear mixed model, we will do the same assumption checks as described in point 5.1.4.

5.2.5 Sub-group Analyses

We will do the following subgroup analyses in the FAS to investigate whether the treatment effect on secondary outcomes differs for certain subgroups. The analysis will be performed by adding the respective variable to the treatment*time interaction in the mixed model described as primary analysis:

Paper 1:

Mental fatigue

- Sex (male, female)
- Age at inclusion (<median, ≥median)
- Years from diagnosis (<median, ≥median)
- Living with children (yes, no)
- Fulltime work (yes, no)
- Education (high, low)
- Number of chemotherapy cycles (2-4, >4 cycles)
- Lymphoma subtype (Hodgkin, non-Hodgkin)
- Radiation therapy (yes, no)
- Baseline level of mental fatigue (divided into tertiles)
- Adherence to the total intervention (completing ≥ 70 % of the exercise program and 65 % of the nutrition counselling and CBT-sessions versus not)
- Adherence to the CBT-sessions (completing ≥ 65%, i.e. four of six sessions versus not)
- Adherence to the exercise program (completing ≥ 70 % of the exercise program versus not)

Physical fatigue

- Sex (male, female)
- Age at inclusion (<median, ≥median)
- Years from diagnosis (<median, ≥median)
- Living with children (yes, no)
- Fulltime work (yes, no)
- Education (high, low)
- Number of chemotherapy cycles (2-4, >4 cycles)
- Lymphoma subtype (Hodgkin, non-Hodgkin)
- Radiation therapy (yes, no)
- Baseline level of physical fatigue (divided into tertiles)
- Adherence to the intervention (completing ≥ 70 % of the exercise program and 65 % of the nutrition counselling and CBT-sessions versus not)
- Adherence to the CBT-sessions (completing ≥ 65%, i.e. four of six sessions versus not)
- Adherence to the exercise program (completing ≥ 70 % of the exercise program versus not)

Følsomhet Intern (gul)

Global quality of life

- Sex (male, female)
- Age at inclusion (<median, ≥median)
- Years from diagnosis (<median, ≥median)
- Living with children (yes, no)
- Fulltime work (yes, no)
- Education (high, low)
- Number of chemotherapy cycles (2-4, >4 cycles)
- Lymphoma subtype (Hodgkin, non-Hodgkin)
- Radiation therapy (yes, no)
- Baseline level of total fatigue (divided into tertiles)
- Baseline global quality of life (<median, ≥median)
- Adherence to the total intervention (completing ≥ 70 % of the exercise program and 65 % of the nutrition counselling and CBT-sessions versus not)

In addition, we will do sub-group analyses on the EORTC QLQ-C30 functioning scales, Subjective Vitality Scale, Satisfaction With Life Scale, Diet Index, muscle strength, VO2peak if the effect of the intervention on these outcomes have a $p<0.1$.

Paper 2:

Symptoms of anxiety

- Sex (male, female)
- Age at inclusion (<median, ≥median)
- Years from diagnosis (<median, ≥median)
- Personality traits measured by BFI-20, dichotomized at median score
- Living alone (yes, no)
- Fulltime work (yes, no)
- Education (high, low)
- Number of chemotherapy cycles (2-4, >4 cycles)
- Lymphoma subtype (Hodgkin, non-Hodgkin)
- Radiation therapy (yes, no)
- Extraversion (<median, ≥median)
- Neuroticism (<median, ≥median)
- Agreeableness (<median, ≥median)
- Conscientiousness (<median, ≥median)
- Openness (<median, ≥median)
- Baseline level of anxiety (GAD score no/mild symptoms (0-9), moderate/severe (10-21))
- Baseline level of total fatigue ((< median, ≥median, tertiles)
- Adherence to the intervention (completing ≥ 70 % of the exercise program and 65 % of the nutrition counselling and CBT-sessions versus not)
- Adherence to the CBT- intervention (completing ≥ 65 % of the CBT-sessions versus not)

Symptoms of depression

- Sex (male, female)
- Age at inclusion (<median, ≥median)
- Years from diagnosis (<median, ≥median)
- Living alone (yes, no)
- Fulltime work (yes, no)
- Education (high, low)
- Number of chemotherapy cycles (2-4, >4 cycles)

Følsomhet Intern (gul)

- Lymphoma subtype (Hodgkin, non-Hodgkin)
- Radiation therapy (yes, no)
- Extraversion (<median, ≥median)
- Neuroticism (<median, ≥median)
- Agreeableness (<median, ≥median)
- Conscientiousness (<median, ≥median)
- Openness (<median, ≥median)
- Baseline level of depressive symptoms (PHQ-9 score no/mild symptoms (0-9), moderate/severe (10-27))
- Baseline level of total fatigue (< median, ≥median or tertiles)
- Adherence to the intervention (completing ≥ 70 % of the exercise program and 65 % of the nutrition counselling and CBT-sessions versus not)
- Adherence to the CBT- intervention (completing ≥ 65 % of the CBT-sessions versus not)

Paper 3:

Work status

- Sex (male, female)
- Age at inclusion (<median, ≥median)
- Years from diagnosis (<median, ≥median)
- Living with children (yes, no)
- Fulltime work at baseline (yes, no)
- Education (high, low)
- Number of chemotherapy cycles (2-4, >4 cycles)
- Lymphoma subtype (Hodgkin, non-Hodgkin)
- Radiation therapy (yes, no)
- Baseline level of total fatigue (< median, ≥median or tertiles)
- Baseline level of work ability (<median, ≥median)
- Work environment at baseline (psychological demands, decision latitude and social support scores <median, ≥median))
- Adherence to the intervention (completing ≥ 70 % of the exercise program and 65 % of the nutrition counselling and CBT-sessions versus not)

Work ability:

- Sex (male, female)
- Age at inclusion (<median, ≥median)
- Years from diagnosis (<median, ≥median)
- Living with children (yes, no)
- Fulltime work at baseline (yes, no)
- Education (high, low)
- Number of chemotherapy cycles (2-4, >4 cycles)
- Lymphoma subtype (Hodgkin, non-Hodgkin)
- Radiation therapy (yes, no)
- Baseline level of total fatigue (<median, ≥median or tertiles)
- Baseline level of work ability (<median, ≥median)
- Adherence to the intervention (completing ≥ 70 % of the exercise program and 65 % of the nutrition counselling and CBT-sessions versus not)
- Work environment at baseline (psychological demands, decision latitude and social support scores <median, ≥median)

Følsomhet Intern (gul)

Paper 4:

Adherence to Norwegian Food-Based Dietary Guidelines:

Sub-group analyses for paper 4 will be conducted for selected food groups and nutrition advice. The list below may be revised in a later SAP version:

- Sex (male, female)
- Age at inclusion (<median, ≥median)
- Years from diagnosis (<median, ≥median)
- Living with children (yes, no)
- Living alone (yes, no)
- Current smoking (yes, no)
- Fulltime work (yes, no)
- Education level (high, low)
- Number of chemotherapy cycles (2-4, >4 cycles)
- Lymphoma subtype (Hodgkin, non-Hodgkin)
- Radiation therapy (yes, no)
- High versus low baseline level of total fatigue (< median, ≥median or tertiles)
- Diet index at baseline (<median, ≥median)
- Lifestyle index at baseline (<median, ≥median)
- Adherence to the nutrition counselling (completing at least 2/3 of the nutrition counselling versus not)

The stratified treatment effect (marginal mean difference at T1 with 95% CIs and p-value of the interaction) by subgroup will be presented, e.g. by using a forest plot.

5.3 Methods for Categorical Secondary Outcomes

Change in work status will be analysed using a mixed effects logistic regression. Alternative analyses with work proportion as outcome will be analysed with mixed effects linear regression, or alternatively beta regression if assumptions are not met.

6 Safety Analyses

General safety evaluations will be based on the incidence, intensity and type of adverse events (AEs).

6.1 Adverse Events

Adverse events will be reported by using the Common Terminology Criteria for Adverse Events (CTCAE) 5th Edition. For tabulations, only testing and/or treatment emerging AEs will be presented. These are defined as AEs with a start date on or after date of randomization.
We will report the following numbers (%) by ITT study group allocation: AEs per person, AE grade, AE relationship to exercise intervention/physical testing, AE leading to study withdrawal and serious AEs.

7 Statistical Software

Statistical analyses will be done in Stata v16 or newer (StataCorp. 2015. *Stata Statistical Software: Release 16*. College Station, TX, USA), R v4.0.2 or newer (R Foundation for Statistical Computing. 2019. *R Development Core Team. Vienna, Austria*) or IBM SPSS Statistics version 29.0 or newer (SPSS, Chicago, IL).

Følsomhet Intern (gul)

8 References

1. Chalder, T., et al., *Development of a fatigue scale.* Journal of Psychosomatic Research, 1993. **37**(2): p. 147-153.
2. Fukuda, K., et al., *The chronic fatigue syndrome: A comprehensive approach to its definition and study.* Annals of Internal Medicine, 1994. **121**(12): p. 953-959.
3. Loge, J.H., et al., *Hodgkin's disease survivors more fatigued than the general population.* J Clin Oncol, 1999. **17**(1): p. 253-61.
4. Reinertsen, K.V., et al., *Predictors and course of chronic fatigue in long-term breast cancer survivors.* Journal of cancer survivorship : research and practice, 2010. **4**(4): p. 405-414.
5. Smeland, K.B., et al., *Chronic fatigue is highly prevalent in survivors of autologous stem cell transplantation and associated with IL-6, neuroticism, cardiorespiratory fitness, and obesity.* Bone Marrow Transplantation, 2018.
6. Sprauten, M., et al., *Chronic fatigue in 812 testicular cancer survivors during long-term follow-up: increasing prevalence and risk factors.* Ann Oncol, 2015. **26**(10): p. 2133-40.
7. Curt, G.A., et al., *Impact of cancer-related fatigue on the lives of patients: new findings from the Fatigue Coalition.* Oncologist, 2000. **5**(5): p. 353-60.
8. Bootsma, T.I., et al., *Experiencing and responding to chronic cancer-related fatigue: A meta-ethnography of qualitative research.* Psychooncology, 2020. **29**(2): p. 241-250.
9. Djalilova, D., et al., *Policy analysis of access to and reimbursement for nonpharmacologic therapies for cancer-related fatigue.* Public Health Nursing, 2019. **36**(4): p. 545-550.
10. Carlotto, A., et al., *The Economic Burden of Toxicities Associated with Cancer Treatment: Review of the Literature and Analysis of Nausea and Vomiting, Diarrhoea, Oral Mucositis and Fatigue.* PharmacoEconomics, 2013. **31**(9): p. 753-766.
11. Cancer Registry of Norway., *Cancer in Norway 2023 - Cancer incidence, mortality, survival and prevalence inNorway.* . 2024, Cancer Registry of Norway. Norwegian Institue of Public Health.: Oslo.
12. Daniels, L.A., et al., *Chronic fatigue in Hodgkin lymphoma survivors and associations with anxiety, depression and comorbidity.* Br J Cancer, 2014. **110**(4): p. 868-74.
13. Schaapveld, M., et al., *Second Cancer Risk Up to 40 Years after Treatment for Hodgkin's Lymphoma.* N Engl J Med, 2015. **373**(26): p. 2499-511.
14. Aleman, B.M., et al., *Late cardiotoxicity after treatment for Hodgkin lymphoma.* Blood, 2007. **109**(5): p. 1878-86.
15. Eikeland, S.A., et al., *Chemotherapy-induced peripheral neuropathy after modern treatment of Hodgkin's lymphoma; symptom burden and quality of life.* Acta Oncologica, 2021. **60**(7): p. 911-920.
16. Eikeland, S.A., et al., *Chronic fatigue in long-term survivors of Hodgkin's lymphoma after contemporary risk-adapted treatment.* Acta Oncologica, 2023. **62**(1): p. 80-88.
17. Mustian, K.M., et al., *Comparison of Pharmaceutical, Psychological, and Exercise Treatments for Cancer-Related Fatigue: A Meta-analysis.* JAMA oncology, 2017. **3**(7): p. 961-968.
18. Bower, J.E., *Cancer-related fatigue: Mechanisms, risk factors, and treatments.* Nature reviews. Clinical oncology, 2014. **11**(10): p. 597-609.
19. Abrahams, H.J.G., et al., *Risk factors, prevalence, and course of severe fatigue after breast cancer treatment: a meta-analysis involving 12 327 breast cancer survivors.* Annals of Oncology, 2016. **27**(6): p. 965-974.
20. Bower, J.E., et al., *Fatigue in breast cancer survivors: occurrence, correlates, and impact on quality of life.* J Clin Oncol, 2000. **18**(4): p. 743-53.
21. Broeckel, J.A., et al., *Characteristics and correlates of fatigue after adjuvant chemotherapy for breast cancer.* J Clin Oncol, 1998. **16**(5): p. 1689-96.

Følsomhet Intern (gul)

22. George, S.M., et al., *Better postdiagnosis diet quality is associated with less cancer-related fatigue in breast cancer survivors.* J Cancer Surviv, 2014. **8**(4): p. 680-7.
23. Bower, J.E., et al., *Management of Fatigue in Adult Survivors of Cancer: ASCO–Society for Integrative Oncology Guideline Update.* Journal of Clinical Oncology, 2024: p. JCO.24.00541.
24. Belloni, S., et al., *A Systematic Review of Systematic Reviews and Pooled Meta-Analysis on Psychosocial Interventions for Improving Cancer-Related Fatigue.* Semin Oncol Nurs, 2023. **39**(3): p. 151354.
25. Haussmann, A., et al., *Meta-Analysis of Randomized Controlled Trials on Yoga, Psychosocial, and Mindfulness-Based Interventions for Cancer-Related Fatigue: What Intervention Characteristics Are Related to Higher Efficacy?* Cancers, 2022. **14**(8): p. 2016.
26. Oberoi, S., et al., *Physical activity reduces fatigue in patients with cancer and hematopoietic stem cell transplant recipients: A systematic review and meta-analysis of randomized trials.* Critical Reviews in Oncology/Hematology, 2018. **122**: p. 52-59.
27. Kessels, E., O. Husson, and C.M. van der Feltz-Cornelis, *The effect of exercise on cancer-related fatigue in cancer survivors: a systematic review and meta-analysis.* Neuropsychiatr Dis Treat, 2018. **14**: p. 479-494.
28. Belloni, S., C. Arrigoni, and R. Caruso, *Effects from physical exercise on reduced cancer-related fatigue: a systematic review of systematic reviews and meta-analysis.* 2021.
29. Twomey, R., et al., *Physical activity after cancer treatment in adults with cancer-related fatigue: a systematic review of randomized trials with fatigue as an eligibility criterion*. 2020.
30. Oldervoll, L.M., et al., *Exercise reduces fatigue in chronic fatigued Hodgkins disease survivors—results from a pilot study.* European Journal of Cancer, 2003. **39**(1): p. 57-63.
31. Bøhn, S., et al., *The feasibility of a multidimensional intervention in lymphoma survivors with chronic fatigue.* Support Care Cancer, 2023. **32**(1): p. 22.
32. Osoba, D., et al., *Interpreting the significance of changes in health-related quality-of-life scores.* J Clin Oncol, 1998. **16**(1): p. 139-44.
33. Nordin, Å., et al., *Minimal important differences for fatigue patient reported outcome measures-a systematic review.* BMC medical research methodology, 2016. **16**: p. 62-62.
34. Åsvold, B.O., et al., *Cohort Profile Update: The HUNT Study, Norway.* International Journal of Epidemiology, 2023. **52**(1): p. e80-e91.
35. Engvik, H. and S.-E. Clausen, *Norsk kortversjon av big five inventory (BFI-20).* Tidsskrift for norsk psykologforening, 2011. **48**(9): p. 869-872.
36. Theorell, T. and R.A. Karasek, *Current issues relating to psychosocial job strain and cardiovascular disease research.* J Occup Health Psychol, 1996. **1**(1): p. 9-26.
37. Godin, G., J. Jobin, and J. Bouillon, *Assessment of leisure time exercise behavior by self-report: a concurrent validity study.* Can J Public Health, 1986. **77**(5): p. 359-62.
38. *Nutrition and Physical Activity Guideline for Cancer Survivors.* CA: A Cancer Journal for Clinicians, 2022. **72**(3): p. 263-265.
39. Aaronson, N.K., et al., *The European Organization for Research and Treatment of Cancer QLQ-C30: a quality-of-life instrument for use in international clinical trials in oncology.* J Natl Cancer Inst, 1993. **85**(5): p. 365-76.
40. *EuroQol--a new facility for the measurement of health-related quality of life.* Health Policy, 1990. **16**(3): p. 199-208.
41. Garratt, A.M., et al., *EQ-5D-5L value set for Norway: a hybrid model using cTTO and DCE data.* Quality of Life Research, 2024.
42. Pavot, W. and E. Diener, *Review of the Satisfaction With Life Scale.* Psychological Assessment - PSYCHOL ASSESSMENT, 1993. **5**: p. 164-172.
43. Ryan, R.M. and C. Frederick, *On energy, personality, and health: subjective vitality as a dynamic reflection of well-being.* J Pers, 1997. **65**(3): p. 529-65.

Følsomhet Intern (gul)

44. Spitzer, R.L., et al., *A brief measure for assessing generalized anxiety disorder: the GAD-7.* Arch Intern Med, 2006. **166**(10): p. 1092-7.
45. Kroenke, K., R.L. Spitzer, and J.B. Williams, *The PHQ-9: validity of a brief depression severity measure.* J Gen Intern Med, 2001. **16**(9): p. 606-13.
46. Tuomi, K., et al., *Work ability index*. Vol. 19. 1998: Finnish Institute of Occupational Health Helsinki.
47. Henriksen, H.B., et al., *A Short Digital Food Frequency Questionnaire (DIGIKOST-FFQ) Assessing Dietary Intake and Other Lifestyle Factors Among Norwegians: Qualitative Evaluation With Focus Group Interviews and Usability Testing.* JMIR Form Res, 2022. **6**(11): p. e35933.
48. Henriksen, H.B., et al., *Digital Food Frequency Questionnaire Assessing Adherence to the Norwegian Food-Based Dietary Guidelines and Other National Lifestyle Recommendations: Instrument Validation Study.* J Med Internet Res, 2024. **26**: p. e53442.
49. Knudsen, M.D., et al., *Reproducibility and comparison of a digital food frequency questionnaire (DIGIKOST-FFQ) assessing adherence to national diet and lifestyle recommendations.* Food Nutr Res, 2024. **68**.
50. Henriksen, H.B., et al., *Development of the Norwegian diet index and the Norwegian lifestyle index and evaluation in a national survey.* Food Nutr Res, 2023. **67**.
51. Helsedirektoratet, *Forebygging, utredning og behandling av overvekt og fedme hos voksne. Nasjonale retningslinjer for primærhelsetjenesten.* . 2011, Helsedirektoratet: Oslo.
52. Williams, G.C. and E.L. Deci, *Internalization of biopsychosocial values by medical students: a test of self-determination theory.* Journal of personality and social psychology, 1996. **70**(4): p. 767.
53. Loe, H., B.M. Nes, and U. Wisløff, *Predicting VO2peak from Submaximal- and Peak Exercise Models: The HUNT 3 Fitness Study, Norway.* PLOS ONE, 2016. **11**(1): p. e0144873.

Følsomhet Intern (gul)
