# Supplementary material for: Effects of a Multidisciplinary Intervention on Fatigue in Lymphoma Survivors With Chronic Fatigue: Protocol for a Randomized Controlled Trial (REFUEL)
Source: JMIR Res Protoc. 2025 Aug 29;14:e69336. doi: 10.2196/69336 (PMC12432467; doi:10.2196/69336)
Supplement: Multimedia Appendix 7 [file resprot_v14i1e69336_app7.pdf]

	STUDY PERIOD																		
	**Enrolment/ pre-randomization (T0)**	**Randomization**	**Intervention**												**3 months post-randomization (T1)**	**6 months post-randomization (T2)**	**9 months post-randomization (T3)**	**1 year follow-up (T4)**	**2 year follow-up (T5)**
**TIMEPOINT**	-1- -3 w	0	1 w	2 w	3 w	4 w	5 w	6 w	7 w	8 w	9 w	10 w	11 w	12 w	3 months	6 months	9 months	15 months	27 months
**ENROLMENT:**																			
Informed consent	x																		
Eligibility/medical screening	x																		
**INTERVENTION:**																			
Patient education			x																
Physical exercise			x	x	x	x	x	x	x	x	x	x	x	x					
Nutrition counselling				x				x				x							
CBT group sessions					x	x	x	x	x	x									
**ASSESSMENTS:**																			
**PROMS**																			
Fatigue	x														x	x	x	x	x
HRQoL	x														x	x	x	x	x
Mental health	x														x	x	x	x	x
Subjective vitality	x														x	x	x		
Satisfaction with life	x														x	x	x		
Work status	x														x	x	x	x	x
Work ability	x														x	x	x	x	x
Exercise competence	x														x	x	x		
Diet	x														x	x	x		
**Physical fitness**																			
VO2peak	x														x	x	x		
Leg strength	x														x	x	x		
Upper body strength	x														x	x	x		
**Additional measures**																			
PREMS																		x	x
HRQoL among partners	x														x	x	x		
**Background information**																			
Socio-demographic/lifestyle characteristics	x														x	x	x	x	x
Cancer-related information	x																		

W: weeks CBT: cognitive behavioral therapy; PROMS: patient-reported outcome measures; HRQoL: health-related quality of life; PREMS: patient-reported experience measures
