# Supplementary material for: Effects of a Multidisciplinary Intervention on Fatigue in Lymphoma Survivors With Chronic Fatigue: Protocol for a Randomized Controlled Trial (REFUEL)
Source: JMIR Res Protoc. 2025 Aug 29;14:e69336. doi: 10.2196/69336 (PMC12432467; doi:10.2196/69336)
Supplement: Multimedia Appendix 8 [file resprot_v14i1e69336_app8.pdf]

STIFTELSEN DAM

Oslo, 15.10.2020

Resultatet av søknadsbehandlingen offentliggjøres i dag

Vi har gleden av å meddele at prosjektet «Tiltak mot kronisk tretthet etter kreft» i regi av «Kreftforeningen», vil få støtte fra Stiftelsen Dam.

Det er «Kreftforeningen» som vil få utbetalt midlene og som du skal forholde deg til. Ta kontakt med prosjektkoordinator i organisasjonen for mer informasjon om hva som skjer videre.

Legg imidlertid merke til tildelingskommentaren under og eventuelle forbehold.

TILDELINGSKOMMENTAR
Tildelingen er gitt til postdoktorstipendiat hel stilling.

BEVILGNINGEN
Bevilgningen er gitt for hele prosjektet, men utbetales for ett år av gangen. Bevilget beløp er gitt til lønn (inkludert sosiale utgifter) og kr. 70 000 i driftstilskudd. Summen er allerede justert for forventet prisutvikling. Det vil si at du vil få utbetalt den samme summen i hver utbetaling. Merk at kostnader forbundet med åpen publisering ikke dekkes av stiftelsen. Driftstilskuddet er oppjustert for å dekke utgifter forbundet med dette.

ÅPEN FORSKNING
Stiftelsen Dam stiller strenge krav til åpenhet i prosjektene generelt og i forskningsprosjektene spesielt.

Kravene er beskrevet i en egen retningslinje for åpenhet, og omhandler blant annet forhåndsregistrering av alle studier og åpen publisering av resultater. Gjør deg kjent med disse kravene.

SØKNADSBEHANDLINGEN
Det er utlysningen som ligger til grunn for vurderingen. I tillegg beskriver fagutvalgets instruks og vår rutine for søknadsvurdering viktige detaljer i søknadsbehandlingen.
Det er lagt opp til at hver søknad får fem individuelle, uavhengige vurderinger i vårt fagutvalg. Nedenfor ser du hvordan de fire kriteriene er vurdert (på skalaen fra 1-7) av de habile fagutvalgsmedlemmene. I tillegg vurderes søknaden av tre brukerrepresentanter. Denne karakteren brukes for å skille mellom søknader som har samme gjennomsnittskarakter fra fagutvalget. Vi understreker at administrasjonen ikke har mer informasjon å dele om søknadsbehandlingen og at det ikke er anledning til å klage. Du kan ved behov hente frem din søknad i Damnett.

KARAKTERER
I tabellen vises gjennomsnittskarakteren for hvert kriterium og totalt (avrundet til nærmeste halve karakter), i tillegg til laveste og høyeste karakter gitt av utvalgsmedlemmene. Som du ser setter utvalgsmedlemmene ulike karakterer. Det er en vanlig utfordring i søknadsbehandling. Derfor sørger vi for at alle våre søknader får flere uavhengige vurderinger

og bruker gjennomsnittet for å sikre at ikke enkeltvurderinger får for stor betydning. I rapporten "Bedre søknadsbehandling" har vi skrevet om forskningen og argumentene som ligger bak måten vi organisert våre vurderingsprosesser.

Fagutvalget

Kriterium	Snitt	Lavest	Høyest
Soliditet	5.50	5	6
Virkning	5.75	5	6
Gjennomføring	5.25	5	6
Prioriteringer	5.75	5	6
Totalt	5.56	5	6

Brukerrepresentantene

Kriterium	Snitt	Lavest	Høyest
Idé og prosjektdefinering	3.33	3	4
Planlegging og gjennomføring	4.00	3	5
Formidling og implementering	4.00	3	5
Totalt	3.78	3	5

KOMMENTARER: STYRKER

Dersom det mangler kommentarer betyr det at fagutvalgsmedlemmet har meldt seg inhabil for vurdering av søknaden. Alle søknader skal ha minimum tre uavhengige vurderinger.

Fagutvalgsmedlem 1

Prosjektet framstår som en godt planlagt studie med stort potensiale for å generere anvendbar kunnskap. Dette er et felt der det er mange faglige miljøer opererer mer ut fra gode intensjoner enn solid kunnskap, så det er svært positivt at det tas initiativ til å gjennomføre en slik tiltaksstudie.

Fagutvalgsmedlem 2

Fagutvalgsmedlem 3

Spennende (men krevende) intervensjon og argumenteres godt for potensiell nytteverdi. God brukermedvirkning.

Fagutvalgsmedlem 4

Sterk søknad. Nyskapende ved at det bruker randomisert kontrollert studie som forskningsdesign. Samordner og intensiverer oppfølging av pasientgruppen. Viktig forskningsspørsmål. Stor potensiell gevinst både for pasientgruppen og samfunnet, da mange av pasientene sannsynligvis har betydelig redusert livskvalitet og befinner seg utenfor yrkeslivet. Solid gjennomføringsplan. Sterk forskningsgruppe.

Ved å lese prosjektbeskrivelsen får man inntrykk av at prosjektet er sterkt forankret i klinisk erfaring – hos behandlere som har ansvar for en pasientgruppe med store vansker, og som føler stort behov for å heve kvaliteten på tilbudet til disse pasientene.

Fagutvalgsmedlem 5
Pasientperspektiv med fatigue som fokus. RCT design med flere viktige intervensjoner sammen. En god søknad som fremstår gjennomførbart med erfarne forskere på denne kreftgruppen.

Brukerrepresentant 1
Det ble gjennomført fokusgrupper i mai 2019 med kreftoverlevende med kronisk fatigue for å beskrive hvordan det er å leve med dette. To kreftoverlevende med kronisk fatigue samt en pårørende er med som brukerrepresentanter, og det planlegges møter to ganger per år med disse. Det planlegges at disse skal gi innspill til planleggings- og formidlingsfasen.

Brukerrepresentant 2
God beskrivelse av brukermedvirkning knyttet til de to siste vurderingspunktene; god systemtilnærming.

Brukerrepresentant 3
Viktig studie som ser på sammenhengen mellom fatigue og lymfekreft. Bra at det skal være med brukere i studien.

KOMMENTARER: SVAKHETER
Fagutvalgsmedlem 1
Begrunnelsen for de forskjellige komponentene i den sammensatte intervensjonen er ganske overbevisende, bortsett fra for ernæringskomponenten. Et raskt søk tyder på at gjeldende dokumentasjon for at antioksidanter etc. skal ha en innvirkning på fatigue, er svært svak (det er publisert mer solide systematiske oversikter på temaet enn de som det vises til i søknaden). Det virker derfor litt rart at en velger å ta dette inn i intervensjonen. Også fordi dette er krevende tiltak for pasientene, og en risikerer kanskje at pasientene gir opp hele tiltakspakka fordi de ikke klarer/orker å gjennomføre kostholdskomponenten.

Fagutvalgsmedlem 2

Fagutvalgsmedlem 3
Som du skriver er dette en kompleks intervensjon som krever betydelig koordinering. Det gjør det også vanskeligere å si hvilke elementer som bidrar til effekten (men totalpakken kan allikevel evalueres). Potensielt sett kan det være noen komponenter som ikke fungerer så godt og det er vanskeligere å fange opp med et slikt design. Ville et faktorielt design der pakkene var frakoblet gi mer informasjon? Det er også noen utfordringer ved å sammenligne intervensjon med med noe som ikke framstår som noen intervensjon for deltakerne. Hvordan er gjennomførbarheten av mange større/kompleks intervensjon hos personer med utmattelse?

Fagutvalgsmedlem 4
Som i alle studier er det en viss usikkerhet knyttet til rekruttering av deltagere. Kompleksiteten i intervensjonen kan også bli en utfordring, da det kan være deltagere som ikke klarer å gjennomføre alle, eller som faller av.

Fagutvalgsmedlem 5
Lite å utsette på dette. Det er alltid et usikkerhetsmoment om man får inkludert de man planlegger i det tidsrommet man har satt opp. Men RCT-studier er ikke mindre viktig av den grunn.

Brukerrepresentant 1
Uklart hvordan brukermedvirkning sikres i design og gjennomføring av intervensjonen. Litt vag beskrivelse av hvordan de reelt bidrar.

Brukerrepresentant 2
Det er imidlertid vanskeligere å se hvordan brukermedvirkning er beskrevet knyttet til det første vurderingspunktet. Derfor noe ned her.

Brukerrepresentant 3
Det er lite aktiv brukermedvirkning i denne studien. Anbefaler å se hvordan erfaringskunnskapen kan brukes mer involverende i studien.

Hilsen Stiftelsen Dam
